# Evolutionary Trajectories of New Duplicated and Putative De Novo Genes

**DOI:** 10.1093/molbev/msad098

**Published:** 2023-05-04

**Authors:** José Carlos Montañés, Marta Huertas, Xavier Messeguer, M Mar Albà

**Affiliations:** Evolutionary Genomics Group, Research Programme on Biomedical Informatics, Hospital del Mar Medical Research Institute (IMIM), Barcelona, Spain; Evolutionary Genomics Group, Research Programme on Biomedical Informatics, Hospital del Mar Medical Research Institute (IMIM), Barcelona, Spain; Computer Sciences Department, Universitat Politècnica de Catalunya, Barcelona, Spain; Evolutionary Genomics Group, Research Programme on Biomedical Informatics, Hospital del Mar Medical Research Institute (IMIM), Barcelona, Spain; Catalan Institution for Research and Advanced Studies (ICREA), Barcelona, Spain

**Keywords:** gene duplication, de novo gene, phylogeny, gene family

## Abstract

The formation of new genes during evolution is an important motor of functional innovation, but the rate at which new genes originate and the likelihood that they persist over longer evolutionary periods are still poorly understood questions. Two important mechanisms by which new genes arise are gene duplication and de novo formation from a previously noncoding sequence. Does the mechanism of formation influence the evolutionary trajectories of the genes? Proteins arisen by gene duplication retain the sequence and structural properties of the parental protein, and thus they may be relatively stable. Instead, de novo originated proteins are often species specific and thought to be more evolutionary labile. Despite these differences, here we show that both types of genes share a number of similarities, including low sequence constraints in their initial evolutionary phases, high turnover rates at the species level, and comparable persistence rates in deeper branchers, in both yeast and flies. In addition, we show that putative de novo proteins have an excess of substitutions between charged amino acids compared with the neutral expectation, which is reflected in the rapid loss of their initial highly basic character. The study supports high evolutionary dynamics of different kinds of new genes at the species level, in sharp contrast with the stability observed at later stages.

## Introduction

The formation of new genes is an important source of evolutionary novelty, which contributes to the adaptation of species to the environment. Mechanisms by which new genes can be generated include gene duplication and de novo gene birth (Ranz and Parsch 2012; Long et al. 2013; Andersson et al. 2015). Single genes can be duplicated by unequal crossing over during meiosis or by mRNA retrotransposition (Prince and Pickett 2002; Kaessmann et al. 2009). Whereas the majority of the new copies are likely to rapidly become pseudogenized, others will be preserved and continue to evolve under negative selection (Innan and Kondrashov 2010). Over time, the new copies can acquire novel functionalities and expression patterns (Ohno 1970; Lynch and Conery 2000). In contrast, de novo genes emerge from previously nongenic sequences of the genome (Levine et al. 2006; Toll-Riera et al. 2009; Knowles and Mclysaght 2009; Tautz and Domazet-Lošo 2011). Pervasive transcription and translation of the genome provide the required raw material for de novo gene origination (Carvunis et al. 2012; Neme and Tautz 2016; Ruiz-Orera et al. 2018; Schmitz et al. 2018). If useful, the new proteins might be retained. These proteins tend to be smaller than the average protein (Begun et al. 2007; Zhou et al. 2008; Toll-Riera et al. 2009). This is expected considering that they derive from randomly occurring open reading frames (ORFs), the majority of which are very small when compared with ORFs coding for phylogenetically conserved proteins (Dinger et al. 2008). Small proteins are often missed when using computational annotation pipelines (Saghatelian and Couso 2015), and this has hampered the identification of de novo originated proteins. More recently, the use of transcriptomics and ribosome profiling data has been used to uncover many new putative de novo genes in different species (Neme and Tautz 2016; Ruiz-Orera et al. 2018; Schmitz et al. 2018; Durand et al. 2019; Zhang et al. 2019; Blevins et al. 2021; Sandmann et al. 2023).

Due to their noncoding origin, recently originated de novo genes have a number of peculiarities with respect to other genes. In addition to being small, the ORFs tend to show a nonoptimal codon usage bias (Toll-Riera et al. 2009; Carvunis et al. 2012; Schmitz et al. 2018; Blevins et al. 2021), which might be associated with lower translation efficiencies (Durand et al. 2019). Additionally, the new proteins tend to be positively charged, at least in yeast and mammals (Papadopoulos et al. 2021; Blevins et al. 2021). Another reported effect of their provenance is an enrichment in transmembrane domains (Vakirlis, Acar, et al. 2020). In contrast, duplicated genes arise from copies of other existing genes, and thus their sequence and structural properties will be initially similar to those of their ancestors.

In general, gene duplication and de novo gene origin have been studied independently, and for this reason, our understanding of the similarities and differences between the two mechanisms of gene origination remains limited. It has been previously noted that species-specific proteins are unexpectedly abundant when compared with new proteins originated at deeper branches (Neme and Tautz 2013; Palmieri et al. 2014; Schmitz et al. 2018; Heames et al. 2020); because the number of genes per species is relatively constant within a lineage, this would indicate that younger genes have a higher propensity to be lost (Palmieri et al. 2014). Since duplicated proteins have sequences and structures already associated with cellular functions, their retention rates could be expected to be higher than those of de novo evolved proteins (Rödelsperger et al. 2019; Bornberg-Bauer et al. 2021). However, whether this is the case remains an open question. Recently emerged de novo genes show high evolutionary rates when compared with more conserved genes (Toll-Riera et al. 2009; Carvunis et al. 2012; Heames et al. 2020); in the case of gene duplicates, a tendency for evolutionary rates to accelerate following the duplication event has also been documented (Force et al. 1999; Pegueroles et al. 2013; Pich I Roselló and Kondrashov 2014). However, these effects have not been directly compared. Thus, it is currently unclear if the initial relaxation of constraints is of a similar magnitude in the two cases or if the subsequent changes in the rate and mode of evolution of the proteins show any similarities. In order to shed light into these questions, here we compare the properties of proteins originated by gene duplication and de novo in phylogenies of yeasts and flies.

## Results

### Identifying Gene Birth Events

**Fig. 1.**
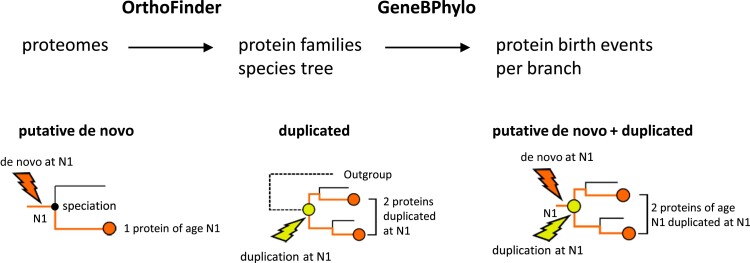
Identification of duplicated and putative de novo gene birth events. The first step is based on running OrthoFinder on a set of proteomes for a given group of species. This generates protein families (orthogroups), branch-specific evolutionary rate estimates, and annotation of paralogous proteins originated at specific branches. The second step, GeneBPhylo, processes the information to identify gene duplication and putative de novo events, and the resulting proteins, originated at each branch in the species tree. Examples of putative de novo, duplicated, and putative de novo + duplicated events are given. N1 refers to the branch in which the event takes place in these examples. A speciation event giving rise to two contemporary species follows. De novo and gene duplication events are indicated with arrows. In the case of putative de novo + duplicated, the graph shows a de novo gene birth event followed by duplication of the gene.

We developed a novel strategy to be able to estimate both gene duplication and de novo gene emergence events in a well-defined species tree, which was based on the program OrthoFinder (Emms and Kelly 2019). OrthoFinder clusters proteins into families on the basis of sequence similarity using BLASTP and then uses a duplication–loss–coalescence (DLC) approach to identify orthologous and paralogous proteins and to estimate the branches at which duplications have occurred. The information provided by OrthoFinder was further processed and integrated using a purpose-built program called GeneBPhylo (fig. 1). Given a reference species, this program generates a list of gene duplication and putative de novo events and the proteins derived from each event. Processing of the data includes the normalization of the number of events inferred in each branch per the branch length (expressed as amino acid substitution rates), so that the rates of formation of new proteins on the different branches can be compared on an equal basis. Proteins found in only the reference species or in a restricted set of species according to the orthogroup species information provided by OrthoFinder are labeled putative de novo proteins (fig. 1). Proteins found to be paralogous to other proteins by the program are defined as duplicated proteins. Putative de novo proteins that have subsequently duplicated are a third class of proteins (fig. 1 putative de novo + duplicated and supplementary fig. S1, Supplementary Material online).

We applied this pipeline to two distinct groups of organisms, yeast and flies. In the first case the reference species was *Saccharomyces cerevisiae* (baker's yeast) and, in the second case, the fruit fly *Drosophila melanogaster*. These are well-annotated, extensively studied species, for which the genomes of close relatives have also been sequenced and annotated, allowing close evolutionary comparisons. To build the tree and protein families, we used the proteomes of 11 yeast species and of 16 insect species (supplementary figs. S2 and S3, Supplementary Material online, respectively). Because our aim was to compare events affecting one or a few genes at a time, we discarded any genes that originated in a previously described whole-genome duplication prior to the diversification of the *Saccharomyces* group (Kellis et al. 2004; Byrne and Wolfe 2005). We also eliminated putative de novo genes that had homologues in more distant species outside the clade (supplementary table S1, Supplementary Material online), to minimize the number of misclassified cases due to multiple gene loses within the clade. In order to avoid redundancies, we did not consider de novo genes that had subsequently duplicated when comparing the properties of putative de novo and duplicated proteins.

### New Genes in *S. cerevisiae*

**Fig. 2.**
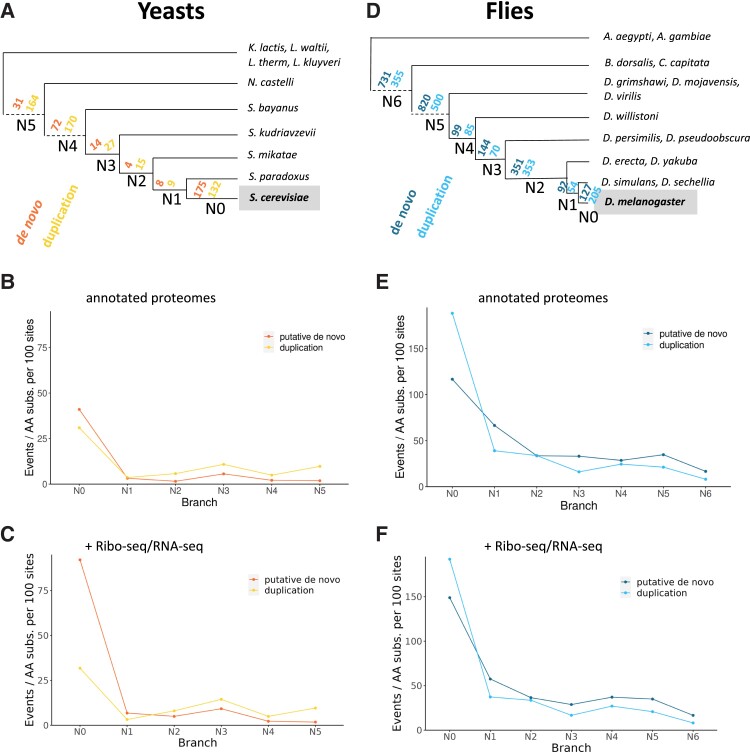
Rate of gene birth and retention in yeast and flies. (*A*) Phylogenetic tree of the yeast clade and number of events per branch. The tree is shown in a schematic way; see supplementary figure S2, Supplementary Material online for a tree with variable branch lengths. In the analysis of new gene birth events, *S. cerevisiae* was taken as the reference species. In addition to the species indicated, *Schizosaccharomyces pombe* was part of the analysis as an outgroup. The estimated number of putative de novo and duplication events at each branch is shown. The information is also provided in supplementary table S2, Supplementary Material online. (*B*) Normalized gene birth events in yeast. The graph shows the number of events in a branch divided by the number of amino acid substitutions per 100 amino acids in the branch. (*C*) Gene birth events in yeast including RNA-Seq/Ribo-Seq ORF predictions. Number of events gene birth events when including new predicted proteins in *S. cerevisiae* using ribosome profiling data as well as in silico translation of novel nonannotated transcripts from newly assembled transcriptomes for the other species (see supplementary table S4, Supplementary Material online for values). (*D*) Phylogenetic tree of the insect clade and number of events per branch. The tree is shown in a schematic way; see supplementary figure S3, Supplementary Material online for a tree with variable branch lengths. In the analysis of new gene birth events, *D. melanogaster* was taken as the reference species. *Tribolium castaneum* was also included in the analysis, but it is an outgroup and therefore not shown. The estimated number of putative de novo and duplication events at each branch is shown. The information is also provided in supplementary table S5, Supplementary Material online. (*E*) Normalized gene birth events in flies. The graph shows the number of events in a branch divided by the number of amino acid substitutions per 100 amino acids in the branch. (*F*) Gene birth events in flies including RNA-Seq/Ribo-Seq predictions. Number of gene birth events when including predicted proteins in *D. melanogaster* using ribosome profiling data as well as in silico translation of newly assembled transcriptomes in eight other *Drosophila* species (see supplementary table S6, Supplementary Material online for values).

In *S. cerevisiae*, we found a large number of gene birth events at the species-specific level (N0), for both de novo and duplicated genes (175 and 132 events, respectively, fig. 2*A* and supplementary table S2, Supplementary Material online). The number of events strongly decreased in subsequent branches of the tree (N1, N2, etc.) for both gene origination mechanisms. The total number of *S. cerevisiae*–specific proteins originated de novo was 192; this value is larger than the number of events (175) because a subset of the proteins had subsequently duplicated. The majority of the putative de novo genes had expression evidence in rich medium (91% with transcripts per million [TPM] > 0.1% and 72% with TPM > 0.5) (supplementary table S2, Supplementary Material online). Among them, we identified *BSC4*, a well-characterized de novo gene with a possible role in DNA repair (Cai et al. 2008). The list also contained YBR196C-A, encoding a protein that integrates into the membrane of the endoplasmic reticulum (Vakirlis, Acar, et al. 2020) and two recently described antisense putative de novo genes, *AUA1* and *VAM10* (Blevins et al. 2021). Among the *S. cerevisiae*–specific duplicated genes, we identified the well-characterized gene pair *CUP1-1*/*CUP1-2*, involved in resistance to high concentrations of copper and cadmium (Fogel and Welch 1982). A previously described example of a duplicated gene pair originated in the common ancestor of *S. cerevisiae*, *Saccharomyces paradoxus*, and *Saccharomyces mikatae* (N2) was *THI21*/*THI22*, encoding a hydroxymethylpyrimidine phosphate (HMP-P) kinase. While *THI21* is required for thiamine biosynthesis, like the ancestral copy THI20, *THI22* is not, indicating rapid functional diversification after gene duplication (Llorente et al. 1999). The vast majority of the putative de novo proteins had no associated Gene Ontology (GO) functions (88%, 169 of 192). Duplicated proteins, on the contrary, were in general annotated. Significantly enriched GO terms included cell wall organization, flocculation, telomere maintenance, and maltose metabolism (false discovery rate < 10^−5^; supplementary material S2, Supplementary Material online).

In a previous work, we defined genomic synteny blocks between pairs of *Saccharomyces* species using clusters of maximum unique matches (MUMs) (Blevins et al. 2021). The synteny blocks are regions that share a common ancestry. Therefore, the majority of de novo genes should be located in regions with conserved synteny. In contrast, regions corresponding to large sequence insertions, such as new gene duplicates, are expected to lack synteny. In accordance, we found that ∼85% of the *S. cerevisiae*–specific genes classified as putative de novo had a syntenic region in *S. paradoxus* (142 out of 166, excluding those which had subsequently duplicated), whereas this value was 56% for the protein duplicates (101 out of 180). We also found that species-specific protein duplicates were frequently found in subtelomeric regions (supplementary fig. S4, Supplementary Material online), in line with the observation that subtelomeric gene families expand much faster than other families (Brown et al. 2010). In contrast, putative de novo genes from the same age, or older gene duplicates (N1–N3), showed no significant clustering in the genome (supplementary fig. S5, Supplementary Material online).

**Fig. 3.**
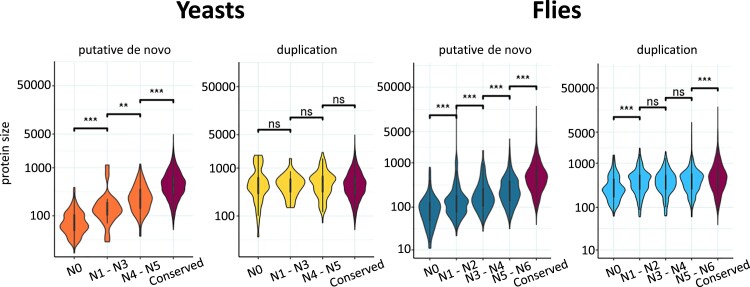
Younger de novo proteins are smaller. Proteins are from *S. cerevisiae* (yeasts) and *D. melanogaster* (flies), classified according to the branch of origin. Conserved: proteins conserved in species outside the clade according to homology searches and not originated by gene duplications in the corresponding tree. The size of putative de novo proteins increases as we consider older branches, for both *S. cerevisiae* and *D. melanogaster*. In *S. cerevisiae*, duplicated proteins show no differences depending on the age. Instead, in *D. melanogaster*, duplicated proteins from N0 tend to be significantly smaller than proteins from older branches. Mann–Whitney–Wilcoxon tests were performed to compare contiguous groups in the graph; significance is denoted as **P < 10^−2^ and ***P < 10^−3^. The number of analyzed proteins is indicated in supplementary tables S2 and S5, Supplementary Material online (*S. cerevisiae* and *D. melanogaster*, respectively); sizes for all proteins in the different groups can be found in supplementary material S2, Supplementary Material online.

Recently emerged de novo genes are expected to be small because of the short size of randomly occurring ORFs. Accordingly, the median size of *S. cerevisiae*–specific proteins was 66 amino acids, compared with 437 amino acids for duplicated proteins of the same age (fig. 3 and supplementary table S3, Supplementary Material online). In the case of de novo genes, the length gradually increased as we considered older branches. In contrast, no significant differences were found for duplicated genes born at different branches of the tree.

The excess of gene birth events at N0, when compared with other branches, became even more evident when we normalized the number of events by the branch length (fig. 2*B*). We observed a sharp decline in the number of events at N1 with respect to N0, for both duplicated and putative de novo genes. The proportion of proteins at N1 compared with N0 was not significantly different between the two types of proteins (chi-square test). However, for branches N2 onwards, we observed that the number of duplication events was approximately double than the number of putative de novo events, pointing to a tendency of duplicated genes to be retained at higher rates in this group.

Some recently evolved genes, especially if arisen de novo, may not be present in the species gene annotations. This is because annotations are often based on the detection of ORFs longer than 100 amino acids and/or with clear homology to other proteins (Yandell and Ence 2012). To better understand the effect of the possible underannotation of small proteins, we performed again the analyses but considered two additional sets of data: 260 novel ORFs with evidence of translation on the basis of ribosome profiling data in *S. cerevisiae* (Blevins et al. 2021) and virtual translations of RNA-Seq–based transcript assemblies of all species except *S. cerevisiae*. With this new data, the number of putative de novo gene births at N0, but also in branches N1–N3, approximately doubled (fig. 2*C*; supplementary table S4, Supplementary Material online compared with supplementary table S2, Supplementary Material online). In contrast, as expected, the effect was very minor for duplicated genes. Thus, the real number of recently evolved de novo genes might be at least twice the number inferred when using the gene annotations alone.

### New Genes in *D. melanogaster*

We applied the same pipeline to *D. melanogaster* and 15 other insect species, including ten extensively characterized *Drosophilae* species (Drosophila 12 Genomes Consortium et al. 2007) (fig. 2*D*). Some of the terminal nodes corresponded to more than one species, detection of a homologous protein in at least one species was considered sufficient to classify the event in the branch connecting the terminal nodes. The number of estimated gene duplication and putative de novo gene birth events in N0 was 205 and 127, respectively (fig. 2*D*). Duplications outnumbered putative de novo gene births in N0 and N1 but not in N2 or in deeper branches. On the basis of the observed values, the retention rate of putative de novo proteins was significantly higher than the retention rate of duplicated proteins (*P* < 10^−10^ when comparing the proportion of genes in N0 vs. N1; chi-square test).

Recently duplicated proteins were enriched in functions related to chromatin structure and transcriptional regulation (supplementary material S2, Supplementary Material online). Instead, putative de novo proteins did not have, in general, known functions. As expected, nearly all de novo genes originated at N0 had a corresponding genomic syntenic region in the *Drosophila simulans* genome (121 out of 122 genes, excluding genes that underwent subsequent duplications), whereas the proportion was much lower for duplicated genes (183 out of 316). As in the case of *S. cerevisiae*, putative de novo protein sequences tended to be longer as we considered more distant branches (fig. 3). In the case of duplicated proteins, those originated at N0 showed a significant tendency to be smaller than proteins originated in other branches. This might be due to partial duplications, which have been reported to be relatively frequent in *D. melanogaster* (Zhang et al. 2022). Comparison of the size of the proteins from the same family indicated that ∼10–15% of the families at N0 might include partial duplications (supplementary fig. S6, Supplementary Material online).

When we normalized the number of events by branch length, we again observed an excess of species-specific events, followed by a rapid decline in N1, and sustained relatively low numbers of proteins in older branches (fig. 2*E* and supplementary table S5, Supplementary Material online). We then predicted novel translated ORFs in *D. melanogaster* using ribosome profiling (Ribo-Seq) data from adult fly heads (Pamudurti et al. 2017) as well as from S2 cells (Douka et al. 2021). A set of 92 putative novel translated products were identified by RibORF (supplementary fig. S7, Supplementary Material online). We investigated if any of these different small ORFs were located in paralogous transcripts, but we only found one case. For comparison, we obtained in silico translations of newly assembled transcriptomes from eight *Drosophila* species (Yang et al. 2018). Running the pipeline with these extended proteomes clearly increased the number of estimated recent de novo gene birth events, especially at N0 and N2 (162–127 and 383–351, respectively), whereas only minor changes were detected for duplication events (fig. 2*F*; supplementary table S6, Supplementary Material online vs. supplementary table S5, Supplementary Material online).

### Relaxation of Selection Constraints after Gene Birth

We next investigated the strength of purifying selection affecting proteins derived from any of the two types of events using single-nucleotide polymorphism (SNP) data. For *S. cerevisiae*, we used SNPs from 1,011 *S. cerevisiae* isolates (Peter et al. 2018) and for *D. melanogaster* data from 192 inbred strains derived from a single outbred population of *D. melanogaster* (Mackay et al. 2012). For different groups of coding sequences (CDS), we calculated the observed ratio of nonsynonymous to synonymous SNPs and divided it to the expected ratio; the latter was estimated by taking into account the species pairwise nucleotide substitution frequencies and the composition of each sequence (Ruiz-Orera et al. 2018). The resulting normalized ratio (PN/PS) measures the strength of purifying selection; the lower the PN/PS value the stronger the purifying selection. Because of the paucity of the SNP data and the short size of the proteins, we merged the information from small adjacent protein groups (e.g., N1 and N2 in flies). The PN/PS for the complete set of CDS was 0.15 in the case of *S. cerevisiae* and 0.1 in the case of *D. melanogaster*, consistent with strong purifying selection in most proteins.

**Fig. 4.**
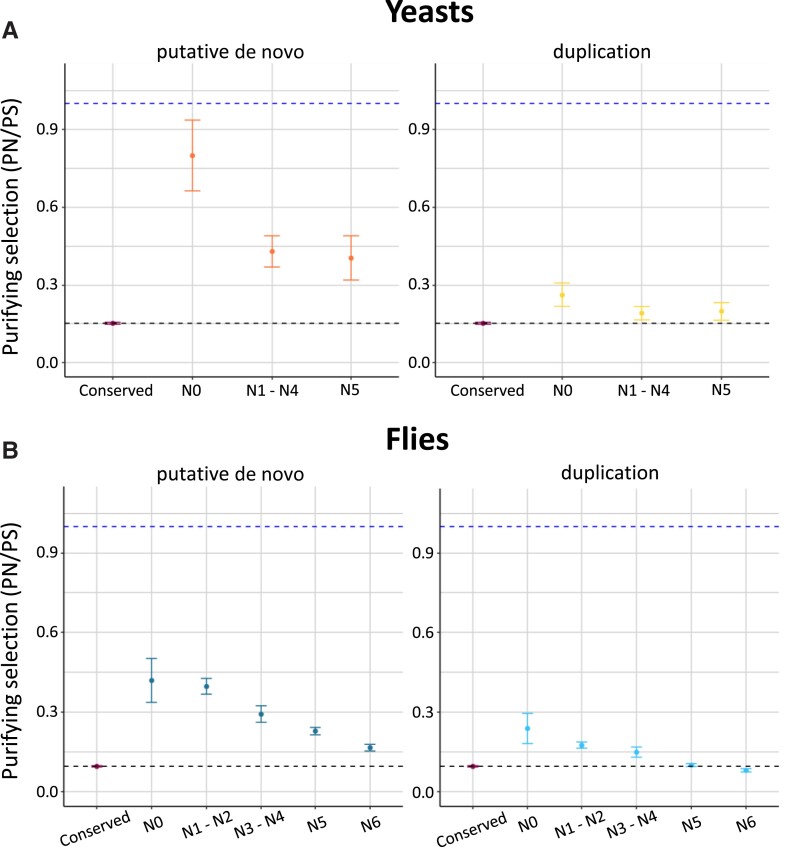
Purifying selection is weaker for young duplicated and putative de novo proteins than that for conserved proteins. (*A*) Yeast proteins. Proteins are classified according to the branch of origin (fig. 2*A*). Conserved refers to proteins with homologs in species outside the clade and not originated by gene duplications in the species tree. (*B*) Fly proteins. Proteins are classified according to the branch of origin (fig. 2*D*). Conserved refers to proteins with homologs in species outside the clade and not originated by gene duplications in the species tree. In both cases, Y axis represents the observed to expected ratio between nonsynonymous substitutions and synonymous substitutions (PN/PS). The expected ratio was estimated using SNPs located in intronic regions. Values ∼1 indicate absence of purifying selection (dashed line). Black dashed line indicates the PN/PS (obs/exp) of all the species genes taken together. Proteins are from *S. cerevisiae* (yeasts) and *D. melanogaster* (flies), classified according to the branch of origin. Conserved: proteins conserved in species outside the clade according to homology searches and not originated by gene duplications in the corresponding tree. Standard deviation for each PN/PS value, shown as vertical lines, was calculated using subsampling (*n* = 1,000) of 1/3 of the genes in each group.

Yeast proteins with a putative de novo origin classified as N0 showed a PN/PS ratio of 0.78, indicating markedly low purifying selection. The PN/PS ratio was around 0.4 in in older proteins from N1 to N4 (fig. 4*A*) (supplementary table S7, Supplementary Material online). This tendency toward increased purifying selection in more phylogenetically conserved proteins is in line with previous observations (Toll-Riera et al. 2009; Carvunis et al. 2012; Ruiz-Orera et al. 2018; Heames et al. 2020). For comparison, the set of proteins derived from gene duplications at N0 had a PN/PS value of 0.26. This value was higher than that observed for older duplicates (0.18–0.19). In *D. melanogaster*, we observed a similar trend of decreasing PN/PS values as we considered older branches, which affected both de novo and duplication events (fig. 4*B*) (supplementary table S7, Supplementary Material online). Although genes with a putative de novo origin at N0 did not display such high PN/PS values as in *S. cerevisiae*, the values were still very high compared with the basal levels (0.4 compared with 0.1). For gene duplicates at N0, the PN/PS value was 0.23, again higher than the basal level. Only the oldest protein duplicates (N5 and N6) had purifying selection levels equivalent to the complete protein data set (∼0.1).

It is well known that gene duplicates tend to evolve in a highly asymmetrical manner (Conant and Wagner 2003; Zhang et al. 2003; Pegueroles et al. 2013; Pich I Roselló and Kondrashov 2014). For this reason, we also calculated PN/PS separately for the fastest and the slowest evolving copy of each gene pair. As before, the values for the fastest evolving copy were highest at N0 and decreased in more distant branches (supplementary fig. S8, Supplementary Material online). In the case of *S. cerevisiae*, the fastest evolving copy at N0 showed a PN/PS of ∼0.43, about four times the basal level. In contrast, in *D. melanogaster*, the fastest evolving copy at N0 showed values that were comparable with the set of putative de novo genes. In conclusion, the data indicated that young duplicated genes can experience a strong relaxation of the selective constraints, which in some cases is comparable with the rates observed for de novo genes.

### Gain of Acidic Amino Acids Over Time

**Fig. 5.**
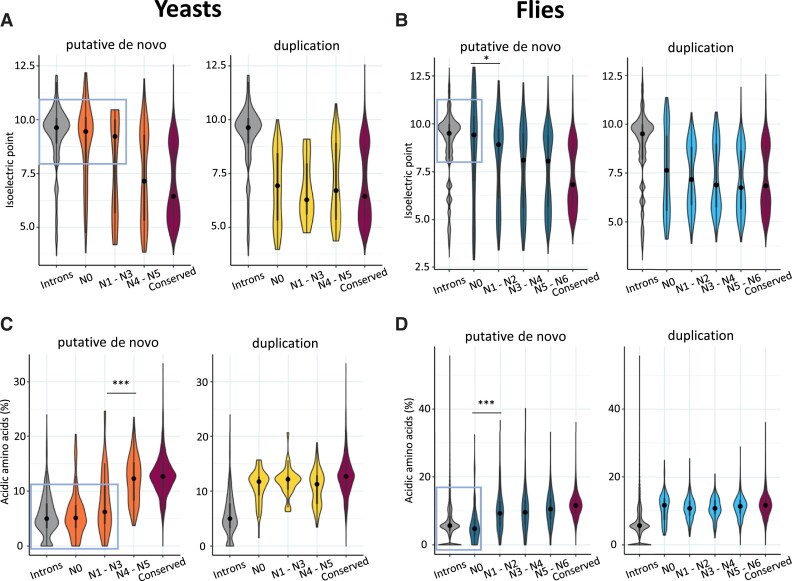
Recently emerged de novo genes are depleted of acidic residues. Charge properties of groups of proteins originated by gene duplication or with a putative de novo origin. Upper figures indicate the isoelectric point (IP) of putative de novo and duplicated genes in yeast (*A*) and flies (*B*). Bottom figures indicate the percentage of acidic or negatively charged amino acids in yeast (*C*) and flies (*D*). Mann–Whitney–Wilcoxon tests were performed to compare contiguous groups in the graph; significance is denoted as *P < 0.05; ***P < 10^−3^.

De novo genes emerge from randomly occurring ORFs in the genome, and this can lead to compositional biases in the nascent proteins (Luis Villanueva-Cañas et al. 2017; Papadopoulos et al. 2021). We examined the amino acid composition and charge of the set of putative de novo proteins and compared it with translated intronic regions, duplicated proteins and a control set of conserved proteins that did not undergo any duplications in the species considered. In both yeasts and flies, we found that recently emerged de novo proteins (N0 to N2 in yeast and N0 in flies) tended to be positively charged, whereas duplicated genes showed no compositional biases with respect to conserved proteins (fig. 5*A*). The high isoelectric point of recently originated de novo proteins was related to a depletion of acidic residues rather than an excess of basic ones (fig. 5*B* and supplementary figs. S9 and S10, Supplementary Material online). Interestingly, nascent de novo proteins had a similar composition than translated noncoding introns (fig. 5). The results are consistent with previous studies in *S. cerevisiae* reporting that recently evolved de novo genes tend to have a high isoelectric point and be depleted of acidic amino acids (Blevins et al. 2021) and that this feature is already present in intergenic ORFs (Papadopoulos et al. 2021). Therefore, the origin of the proteins from noncoding parts of the genome can explain their basic character.

Interestingly, putative de novo proteins originated in a more distant past (from N4 in yeasts and from N1 in flies) did not show a high isoelectric point but were similar to highly conserved proteins. We then hypothesized that negatively charged amino acids might be gained at an abnormally high rate during the first stages of the evolution of the proteins, the alternative explanation being that new basic proteins tend to persist at much lower frequencies than other types of new proteins. To test the hypothesis, we examined the amino acid replacements in sequence alignments of *D. melanogaster* and *D. simulans* proteins, and of *D. melanogaster* and *Drosophila sechellia* proteins, for class N1 as well as for conserved proteins (proteins conserved in the most basal species of the tree and not associated with any gene duplication event). The analysis indicated that there was an excess of basic/acidic pairs in the alignments of the N1 proteins when compared with the conserved ones (supplementary fig. S11 and table S8, Supplementary Material online). Among the changes involving acidic residues, the most common one was lysine/glutamic acid (K/E), which accounted for 17% of the substitutions involving acidic amino acids, compared with ∼9% in the case of conserved proteins (*P* = 0.0024, Fisher test with multiple test correction). The lower number of proteins in yeast when compared with flies (8 vs. 115 classified as N1, respectively) prevented performing a similar analysis in the first group.

Next, we investigated if the bias in the amino acid substitutions occurring in young de novo proteins was expected given the codon frequencies of the set of sequences under study and the species mutational bias. The mutational bias was obtained from intronic SNPs (supplementary fig. S12, Supplementary Material online), and the codon frequencies were calculated separately for N1 and conserved proteins, to take into account any underlying differences between the two groups. For the comparison of the observed versus expected values, we focused on amino acid substitutions that could be explained by a single-nucleotide change, which are the predominant ones given the short phylogenetic distance between the species (79% of the observed changes between *D. melanogaster* and *D. sechellia* N1 proteins and 88% between *D. melanogaster* and *D. simulans* N1 proteins). One example would be substitutions from lysine to glutamic acid, caused by a mutation from A to G (or G to A for glutamic acid to lysine).

**Fig. 6.**
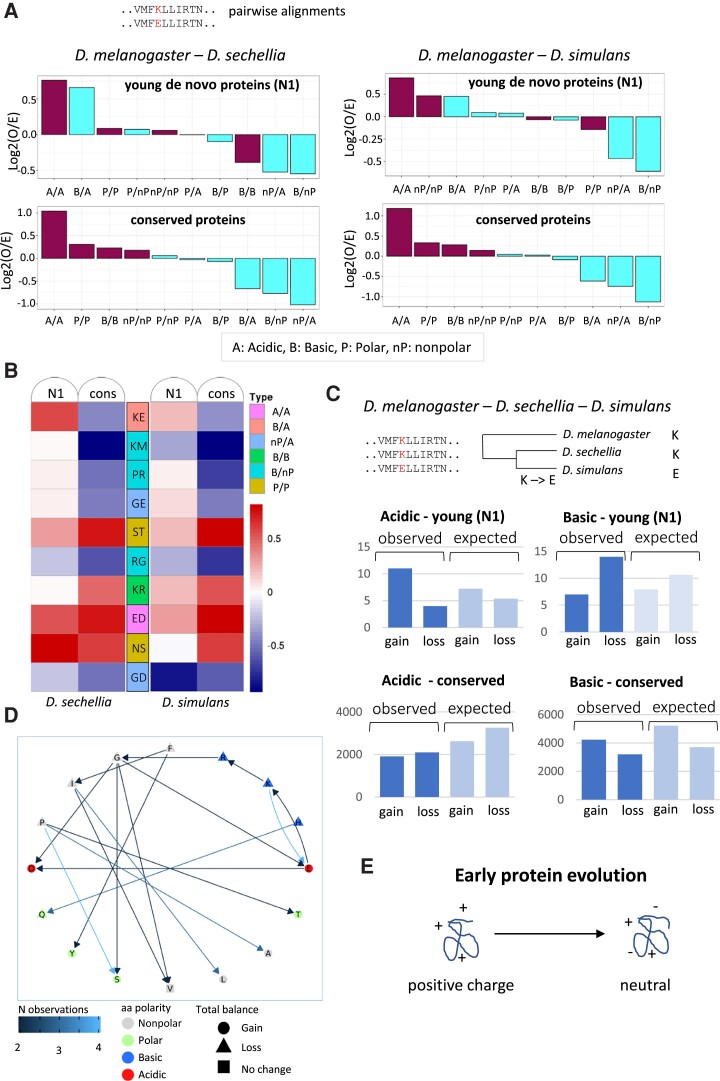
Early evolution of putative de novo proteins is related to gain of negatively charged residues. (*A*) Enrichment of basic/acidic amino acids pairs in pairwise alignments of young proteins. The observed frequency of different amino acid pairs (observed or O) was compared with a null model (expected or E). The logarithm (base 2) of the O/E ratio is represented. Deviations from the null model might indicate selection. (*B*) Observed versus expected frequencies of amino acids pairs. The heat map represents the log_2_ (O/E) values; pairs of amino acids with more than five cases in both *D. melanogaster*—*D. sechellia* and *D. melanogaster*—*D. simulans* protein sequence alignments were selected; for visualization purposes, only the groups acidic/acidic (A/A), basic/acidic (B/A), nonpolar/acidic (nP/A), basic/basic (B/B), basic/nonpolar (B/nP), and polar/polar (P/P), which show the strongest deviation from neutrality in conserved proteins, are displayed. KE pairs are less frequent than expected in conserved proteins but more frequent than expected in N1 proteins, differences in O versus E between the two types of proteins are significant in alignments *D. melanogaster* and *D. sechellia* (chi-square test *P* = 0.0017). (*C*) Acidic residues tend to be gained, and basic residues lost, in the early evolution of proteins. Gain and loss of acidic and basic residues inferred from alignments of orthologous proteins from the three species, for groups N1 and conserved. The number of cases in N1 is relatively small, and the observed biases are not statistically significant. (*D*) Amino acid changes inferred from the three species alignments. N observations refers to the number of changes from one amino acid to another (arrows). The shape of the amino acid indicates if the total number of a specific amino acid decreases, increases, or stays equal (gain is equal to loss). Overall, acidic amino acids (E and D) were gained and basic ones (K, R, and H) were lost. Proline (P) was also lost. (*E*) Model for the increase in the negative charge of young proteins. It includes changes from basic to acidic (e.g., K→E) as well as other acidic amino acid gains (e.g., G→E and G→D).

The comparison of the observed and expected values clearly showed that the alignments of young proteins (N1) contained more basic–acidic pairs than expected by chance (positive log_2_ O/E values in fig. 6*A*; data in Supplementary material 2, Supplementary Material online). This was observed in both alignments of *D. melanogaster* and *D. sechellia* and of *D. melanogaster* and *D. simulans*. In contrast, the same types of changes were less frequent than expected by chance in conserved proteins (negative log_2_ O/E values in fig. 6*A*). Only pairs of amino acids of the same type (acidic/acidic, polar/polar, etc.) had positive log_2_ O/E values in the latter class of proteins.

At the level of specific amino acid changes, we again observed that the frequency of the KE pair in the young proteins was higher than expected by chance, whereas this did not happen in the case of conserved proteins (fig. 6*B*). There were 35 K/E pairs in *D. melanogaster* and *D. sechellia* sequence alignments of young de novo proteins (N1), whereas 23 were expected by chance. In the case of conserved proteins, we observed 1,853 K/E pairs versus 2,872 expected. The differences in observed versus expected between the two groups were statistically significant (chi-square test *P* = 0.0017). Other substitutions involving charged amino acids, such as K/M, P/R, or G/E, were strongly disfavored in conserved proteins but found at frequencies close to the neutral expectation in the case of young proteins.

To gather more details into this process, we inspected the cases in which *D. melanogaster* and one of the sister species—*D. simulans* or *D. sechellia*—shared the same amino acid at a given position, but the other species had a different amino acid. For these cases, one can assume that the shared amino acid is the ancestral one, and this provides information on the direction of the change. For young proteins (N1), we identified 11 gains of an acidic amino acid versus 4 loses and the opposite trend for basic amino acids, 7 gains versus 14 loses (fig. 6*C*). In contrast, the tendencies were reversed in the case of conserved proteins. Part of these differences might be explained by the initial unbalance in the amount of codons for basic and acidic amino acids, but the deviation from the neutral model also points to a possible effect of positive selection. Figure 6*D* shows the different types of amino acid changes that were observed more than once in young proteins, as well as their directionality. All three basic amino acids decreased their frequency, and the two acidic amino acids increased it. Proline residues were also more often lost than gained (12 vs. 3, respectively). Taken together, the observations support the hypothesis that recently emerged de novo proteins tend to gain negatively charged amino acids and become less basic over time (fig. 6*E*).

## Discussion

Species- and lineage-specific genes, which lack homologues in distant organisms, have been a prominent but mysterious feature of newly sequenced genomes (Dujon 1996). Over the past years, evidence has accumulated that a large fraction of them are likely to have originated de novo from previously noncoding genomic regions (Albà and Castresana 2005; Toll-Riera et al. 2009; Tautz and Domazet-Lošo 2011; Zhang et al. 2019; Vakirlis, Carvunis, and McLysaght 2020; Schmitz and Bornberg-Bauer 2017). A previous study in *Drosophila obscura* provided evidence that younger genes are likely to be lost at higher frequencies than more conserved genes (Palmieri et al. 2014). This helped to reconcile observations of a large number of “orphan” species-specific genes (Dujon 1996; Khalturin et al. 2009; Neme and Tautz 2013) with the approximately constant number of genes in a clade. Because duplicated proteins have sequences and structures that have already proven to be useful, they could in principle be more evolutionary stable (Rödelsperger et al. 2019). In a recent study in nematodes (Prabh and Rödelsperger 2022), the researchers observed that de novo protein candidates contributed less to old gene age classes than known proteins families (defined as those in which more than half of the members contained an annotated protein domain). This could mean that de novo candidates were not as evolutionary stable as new genes originating from duplication, which were part of known families. In this work, we have performed a more direct comparison of the number of gene duplication and de novo gene birth events in different branches of the phylogenetic tree. We have observed that, in both cases, there is a peak of species-specific events, which declines sharply when we consider older branches. This means that, independently of the mechanism of origin, the vast majority of the genes formed in a given species are likely to be subsequently lost in the same species lineage. In older branches, the number of events is relatively constant, suggesting that, in contrast, genes that survive beyond the species are rarely lost.

Duplicated and putative de novo proteins showed similar evolutionary trajectories, including an excess of genes at the species-specific level, but had very distinct sequence properties. In the case of de novo proteins, the initial amino acid sequence length was remarkably short, consistent with an origin from randomly occurring ORFs (Albà and Castresana 2005). This class of proteins tended to become progressively longer as we considered more distant branches as time of origination. A possible explanation is that proteins tend to increase in size over evolutionary time, perhaps by the acquisition of new domains, for example, by exon shuffling (Long et al. 2003), or by mutational biases favoring in-frame insertions over deletions (Laurie et al. 2012). We also found that both putative de novo and duplicated proteins experienced a relaxation of the selective constraints after birth, but in the latter case, the effect was more limited in time, with duplicates in the most distant branches showing evolutionary rates similar to conserved proteins. A long-standing question is whether the progressive decrease in the evolutionary rates of putative de novo proteins means that the rates tend to slow down over time (Albà and Castresana 2005; Vishnoi et al. 2010). As a protein evolves and becomes more efficient, changes in the amino acid sequence might tend to be more deleterious and the rate of change decrease. In the case of duplicated proteins, where evolutionary trees with multiple outgroup sequences can be examined, such a decrease in the rates has been observed (Pegueroles et al. 2013; Pich I Roselló and Kondrashov 2014). Studying changes in the evolutionary rates of recently evolved de novo proteins is however more difficult because of the lack of outgroup species. In previous work using both divergence and polymorphism data, rapid evolution of mammalian-specific genes has been related to relaxed purifying selection but not to an increase in the proportion of adaptive substitutions (Gaya-Vidal and Alba 2014). In contrast, recent work using adaptive landscapes has shown that younger proteins in *Drosophila* and *Arabidopsis* are undergoing faster rates of adaptive evolution and tend to accumulate more substitutions with larger physicochemical effects than older proteins (Moutinho et al. 2022).

A large number of recently duplicated genes in *S. cerevisiae* were found in subtelomeric regions. These regions appear to be particularly flexible to accommodate new genes, such as enzymes involved in the degradation of maltose (Brown et al. 2010), which were also detected in our study. Perhaps not surprisingly, copy number variants across different *S. cerevisiae* isolates, as well as horizontally transferred genes, have also been found to be enriched in these regions (Peter et al. 2018). Instead, putative de novo genes did not show any location preference and were found throughout the genome.

We found clear differences in amino acid composition between duplicated and putative de novo proteins. Recently emerged de novo proteins had a marked basic character, which was not observed in young duplicated proteins. In *Drosophila*, an excess of lysine and arginine in small ORFs was previously noted (Couso and Patraquim 2017). Here, we found that the youngest putative de novo proteins had a high isoelectric point, similar to in silico translated intronic sequences. By studying the amino acid changes in alignments of young *Drosophila* proteins, we obtained evidence that they tend to gain acidic amino acids over time. The frequencies at which we observed such changes were above the neutral expectation, which would be consistent with selection playing a role in favoring these particular types of substitutions. A positive charge of the protein could favor the crossing of plasma membranes or interactions with DNA or RNA (Couso and Patraquim 2017). Therefore, a less basic character could reduce the number of unspecific interactions of the protein with cytoplasmic RNA. This, in turn, could provide a selective advantage by increasing the amount of available free protein.

Many of the observations were common to yeast and flies, but there were also a number of differences between the two groups of organisms. In general, the *S. cerevisiae* genome appeared to encode more species-specific de novo proteins than *D. melanogaster*, when compared with other groups of proteins. This might be explained by a longer terminal branch in the former case (0.043 vs. 0.011 substitutions/site), but differences in annotation criteria or completeness could also have played a role. Putative de novo proteins classified as N0 in *D. melanogaster* did not have such extreme PN/PS rates as those in *S. cerevisiae*, perhaps denoting more conservative criteria when annotating the fly proteins. When considering more ancestral branches, the number of gene birth events normalized by branch length was clearly higher in flies than that in yeast. This might be expected if we consider that the former have higher genome complexity—in terms of genome size and number of genes—than the latter.

The number of de novo originated genes in a species varies from study to study (Van Oss and Carvunis 2019). This depends on the starting set of gene annotations and also on the methodology employed to identify possible homologues in other species. For example, in a previous study in baker's yeast, we considered that the detection of gene expression in the equivalent genomic region of another species was sufficient evidence not to consider the gene as species specific (Blevins et al. 2021). But these criteria could include cases in which the transcripts encoded completely unrelated proteins or were noncoding. Instead, here we based our analyses on annotated proteomes, relying on the information provided by OrthoFinder to make further inferences. By doing so, we could study the two mechanisms of gene origination (de novo and duplication) side by side, using the same starting data and a unified pipeline. The number of *S. cerevisiae* putative de novo proteins was relatively similar to that previously reported by Carvunis et al. (2012). Instead, we identified a much larger number of *S. cerevisiae*–specific de novo proteins than Vakirlis et al. (2018), probably because the latter study incorporated an additional filter based on the coding score.

To control for the possible heterogeneity in the gene annotations of different species, we investigated which was the effect of adding ORFs with Ribo-Seq–based evidence of translation, as well as ORFs derived from reconstructed transcriptomes, to the annotations. After running the complete pipeline again, we could observe that the number of putative de novo proteins clearly increased as a result of considering the additional data, denoting that many small proteins still remain to be annotated. The effect in *D. melanogaster* was more modest than that in *S. cerevisiae*, perhaps because many of the de novo genes in flies have been reported to be expressed in testis (Begun et al. 2007; Zhao et al. 2014), and no Ribo-Seq data of sufficient quality were available for this organ.

The estimation of the age of putative de novo genes is not independent of divergence time: Homologues are expected to become more difficult to detect with increasing phylogenetic distance, because of the larger number of accumulated substitutions (Rost 1999; Albà and Castresana 2007; Jain et al. 2019; Weisman et al. 2020). This should barely affect the comparisons of very closely related species but be of relevance when considering long evolutionary distances. For example, using sequence evolution simulations, it has been estimated that, for comparisons of *S. cerevisiae* against the closely related species *S. paradoxus*, *S. mikatae*, or *Saccharomyces kudriavzevii* (branches N1–N3 in the tree we used; see fig. 2*A*), the proportion of misclassified proteins is <5%. For more distant comparisons, however, lack of homology can become more difficult to disentangle from rapid sequence divergence. Vakirlis, Carvunis, and McLysaght 2020 recently developed a method based on genomic synteny blocks to estimate the maximum percentage of true homologues that might be missed using sequence similarity searches alone. They concluded that this fraction was ∼15% for comparisons of *S. cerevisiae* and *Saccharomyces castelli* (equivalent to N5 in our yeast tree; fig. 2*A*) and ∼20% for comparisons of *D. melanogaster* and *Drosophila mojavensis* (N4 in our flies tree; fig. 2*D*). This means that some of the proteins at N4, or more distant branches, could be older than inferred here. Because of these limitations, we have used the term putative de novo proteins (rather than just de novo proteins) throughout the manuscript. However, it is worth noting that, if we were strongly overestimating the number of new genes at the most distant branches (N4–N6), with respect to most recent branches (N1–N3) (where we expect less errors), we should see an increase in the rates of new genes in the former branches, which we do not observe.

Despite being annotated, only a few of the putative de novo proteins had known functions. This can be expected given the lack of conservation in other species. We found that the majority of them were expressed in normal conditions but, without any direct experimental functional evidence, it remains unclear which fraction of the proteins are really functional. In the future, this might be addressed with CRISPR–based functional screenings, as recently been done for a set of human de novo microproteins (Vakirlis et al. 2022). In this study, the authors inspected a large set of small ORFs with translation evidence in several human cell lines (Chen et al. 2020) and identified 155 human de novo originated microproteins. Then, using the results of the CRISPR-Cas screening performed by Chen et al. (2020), they found that 44 of these proteins were likely to be functional. The characterization of the functions of a larger number of de novo proteins will help to understand if these proteins tend to be enriched in particular cellular pathways.

Other limitations of the study are related to the incompleteness of the gene annotations. Because of their small size and lack of phylogenetic conservation, de novo proteins are expected to be poorly annotated. In addition, they are more difficult to detect by proteomics techniques than longer proteins (Ruiz-Orera et al. 2015). Studies using Ribo-Seq data have uncovered many new translated small ORFs (Ingolia et al. 2009; Mudge et al. 2022). However, these data are still relatively scarce; for example, we only found one study with data of sufficiently high quality to annotate translated ORFs for *D. melanogaster* adults. Besides, the data are missing from nonmodel species, preventing direct comparisons of the same kind of data across species. Improving the gene annotations will allow increasing the accuracy of the catalogs of de novo genes in future studies.

This study provides new clues about the evolution of new genes, revealing unexpected similarities between gene duplication and de novo gene birth, despite the differences in the composition and length of the sequences. The excess of new genes in the terminal branches of the tree, regardless of the mechanism of origination, strongly suggests that there is a very high turnover of genes at the level of the species, which has no parallel for genes conserved in more than one species. Future studies at the level of populations might provide useful data to better understand these dynamics and the role of adaptive evolution.

## Materials and Methods

### Annotated Proteins

We extracted the gene annotations from the different species considered in the study from several public resources, including the National Center for Biotechnology Information (O’Leary et al. 2016), Ensembl (Yates et al. 2020) and InsectBase (Yin et al. 2016) (see supplementary tables S9 and S10, Supplementary Material online for a complete list of sequence resources). We used gffread to extract the sequences from the annotated CDS (using -J and -y options). Sequences in which the CDS did not start with an ATG, did not finish with a stop codon, or contained internal stop codons were discarded. We selected the longest protein per gene when several isoforms existed. We also eliminated any proteins that overlapped by >10% of the length of their sequence with another protein sequence encoded on the same genomic strand. The resulting set of annotated proteins was used for all analyses except those described for figure 2*C* and *F* (see below).

### Prediction of Novel Translated ORF Using Ribo-Seq Data

We obtained a set of novel ORFs with translation evidence in *S. cerevisiae* and *D. melanogaster*. In the case of *S. cerevisiae*, we used an already described set of novel proteins that were identified by the analysis of ribosome profiling data with the RibORFv.1.0 software (Blevins et al. 2021). The predictions were based on the observation of significant three nucleotide periodicity and homogeneity of the mapped Ribo-Seq reads. In the case of *D. melanogaster*, we obtained ribosome profiling data from adult fly heads (bioproject PRJNA316472) (Pamudurti et al. 2017) and S2 cells (SRR13664946) (Douka et al. 2021). The Ribo-Seq reads were mapped to a *D. melanogaster* de novo assembled transcriptome (Yang et al. 2018), and translated ORFs were predicted by RibORFv1.0 (Ji et al. 2015). We selected ORFs starting with ATG/TTG/CTG/GTG, longer than 30 nucleotides and a RibORF score ≥0.7. With these cutoffs, we could predict translation of the majority of annotated CDS as well as of 92 nonredundant ORFs in novel transcripts. The novel ORFs with translation evidence were added to the protein annotations for the analyses described in relation to figure 2*C* and *F*.

### In Silico Translation of Nonannotated Transcripts

We generated in silico translated sequences from nonannotated transcripts derived from different de novo assembled transcriptomes for species other than the reference species (see below). In the case of yeast, we used a set of previously obtained transcriptomes that comprised all the species considered here (Blevins et al. 2019). For flies, we used previously published transcriptomes from eight *Drosophila* species: *D. melanogaster*, *Drosophila yakuba*, *Drosophila persimilis*, *Drosophila pseudoobscura*, *Drosophila willistoni*, *Drosophila grimshawi*, *D. mojavensis*, and *Drosophila virilis* (Yang et al. 2018). Additionally, we assembled new transcriptomes for *D. sechellia*, *D. simulans*, and *Drosophila erecta* from available RNA-Seq data (Ma et al. 2018), using the same pipeline employed by Yang et al. 2018. The ORFs were defined from NTG (ATG/CTG/TTG/GTG) to stop codon in frame and encoding at least ten amino acids. These in silico translated sequences were used to investigate the possible existence of nonannotated homologues for the analyses presented in figure 2*C* and *F*.

### Gene Expression

We checked for gene expression in the reference species, both at the level of the transcriptome and translatome, using RNA-Seq and Ribo-Seq data, respectively. In the case of *S. cerevisiae*, we used the data for yeast grown in a rich medium available from Blevins et al. 2021. In the case of *D. melanogaster*, we used the data from Zhang et al. (2018) in 3- to 10-day adult bodies. We mapped the sequencing reads to the annotated transcripts using STAR v2.7.8 (Dobin et al. 2013) and quantified the number of reads mapping to each transcript with featureCounts (Liao et al. 2014). The number of reads per transcript was normalized to TPM.

### Identification of Putative De Novo and Duplication Gene Birth Events

The proteomes of each species were used as input for OrthoFinder (Emms and Kelly 2019). Because we wanted to focus on local gene duplication events, we did not consider *S. cerevisiae* genes previously reported to have arisen from a whole-genome duplication at the basis of the *Saccharomyces* group (Byrne and Wolfe 2005). OrthoFinder clusters the proteins into families (orthogroups), builds phylogenetic trees, and predicts the branches in the tree in which duplication events have taken place. We selected MAFFT (v7.455) for multiple sequence alignments (Katoh and Standley 2013) and IQTree (v1. 6.12) for tree building (Nguyen et al. 2015). Putative de novo gene birth events were identified on the basis of the species distribution within the orthogroups and taking into account the species tree. The most distant species in the orthogroup was used to identify the branch in which the possible origin of the protein had taken place. For example, proteins from families in which there were only proteins from *S. cerevisiae* were classified as N0; those in which there were proteins from *S. cerevisiae* and *S. mikatae*, but not from other species, were classified as N2. Those at N5 were derived from events predicted to have occurred in the branch connecting the *Saccharomyces* and *Lachancea* genus. Additionally, proteins classified as putative de novo were eliminated if possible homologues existed in at least two other species from other groups using BLASTP searches (Altschul et al. 1997) (BlastP *E* < 0.001) (supplementary table S1, Supplementary Material online). The branches at which duplicated events were inferred to have taken place were obtained from the OrthoFinder output. Overall, we defined six proteins classes in yeasts, N0–N5, from more recent to more distant events, and seven classes in Flies, N0–N6, from more recent to more distant events. The branch lengths of the species tree, generated by OrthoFinder using information from all families, were used to normalize the number of events per branch length (number of amino acid substitutions per site). In a small fraction of the families, we identified both putative de novo and duplication events (see details in supplementary tables S2 and S4–S6, Supplementary Material online). When analyzing protein properties, putative de novo proteins which had subsequently duplicated were not taken into account to differentiate more clearly between the features associated with the two mechanisms. We investigated the possible enrichment in particular GO terms (Biological Process) in recently formed proteins (N0) with the software DAVID (Sherman et al. 2022).

### Genomic Synteny Blocks

Genomic synteny blocks between *S. cerevisiae* and *S. paradoxus*, and between *D. melanogaster* and *D. simulans*, were obtained using a previously described approach, based on the identification of clusters of MUMs using a modification of the M-GCAT program (Treangen and Messeguer 2006; Blevins et al. 2021). In this implementation, groups of parallel, consecutive, and neighboring MUMs are clustered into synteny blocks. We used a maximum distance of 100 bases to cluster two consecutive MUMs, for both yeast and flies. We then inspected how many putative de novo and duplicated genes were located in synteny blocks. Because of their noncoding origin, we expect most de novo genes to be located in synteny blocks. Instead, we only expect part of the duplicated genes to map to synteny blocks.

### Purifying Selection Tests Using SNPs

We used published SNPs to assess the strength of purifying selection in different groups of CDS. In the case of *S. cerevisiae*, we used data from 1,011 isolates (Peter et al. 2018). We selected SNPs with a minor allele frequency of at least 5% to minimize the possibility of including mutations under positive selection in one or a few isolates. In *D. melanogaster*, we used the data from 192 inbred strains derived from a single outbred population of *D. melanogaster* known as the *D. melanogaster* genetic reference panel (Mackay et al. 2012). We discarded any sense–antisense overlapping CDS for this analysis, and we did not consider proteins with a putative de novo origin that had subsequently duplicated. Because of the paucity of the SNP data, and the small size of some of the groups (e.g., N1, N2, and N3 in *S. cerevisiae*), we decided to build three representative groups in *S. cerevisiae* (N0, N1–N4, and N5) and five in *D. melanogaster* (N0, N1–N2, N3–N4, N5, and N6). For comparison, we also extracted SNPs from CDS of conserved genes (present in the most basal species of the tree and not associated with subsequent duplication events). The observed SNPs were classified as nonsynonymous (PN), when they altered the amino acid, and as synonymous (PS), when they did not. These values were used to calculate PN/PS(obs) for each group of sequences. We also computed PN/PS(exp) using the species pairwise mutation frequencies (estimated from intronic regions not overlapping any exonic sequence) and the codon composition of the sequences under study (Ruiz-Orera et al. 2018). The ratio between PN/PS (obs) and PN/PS (exp), or normalized PN/PS, provides an estimation of the strength of purifying selection. Values ∼1 are expected in neutrally evolving CDS and values <1 in sequences under purifying selection. To test for significant differences between PN/PS (obs) and PN/PS (exp), we used a Pearson's chi-squared test with Yate's continuity correction and one degree of freedom.

### Amino Acid Composition and Charge

We extracted amino acid frequencies from all *S. cerevisiae* and *D. melanogaster* proteins and clustered them according to their properties (acidic, basic, polar, and nonpolar). Isoelectric point was calculated using the computePI function from the seqinr package in R (Charif et al. 2005). For these analyses, we discarded any proteins initially classified as both putative de novo and duplicated (proteins with a putative de novo origin that had subsequently duplicated).

### Identification of Amino Acid Changes in Sequence Alignments

We extracted amino acid substitutions from the alignments of the proteins in the orthogroups generated by OrthoFinder. We focused on orthogroups containing putative de novo proteins from class N1 and conserved proteins. First, we extracted the data for pairs of species, *D. melanogaster* and *D. sechellia*, and *D. melanogaster* and *D. simulans*, obtaining the frequency of all possible pairs of different amino acids in the alignments. For *D. melanogaster* and *D. sechellia* N1 proteins, we found 718 changes that could be explained by a single-nucleotide substitution (908 in total). For *D. melanogaster* and *D. simulans* alignments, this number was 842 changes (958 in total). We also analyzed alignments containing one protein for each the three species in order to identify substitutions that had occurred after the split of *D. simulans* and *D. sechellia* and for which we could infer the directionality of the change. These were cases in which *D. melanogaster* and *D. simulans* had the same amino acid but *D. sechellia* had a different one (the change would have occurred on the *D. sechellia* branch) or cases in which *D. melanogaster* and *D. sechellia* had the same amino acid but *D. simulans* had a different one (the change would have occurred on the *D. simulans* branch). The latter data set consisted of 86 amino acid changes for N1 and 39,114 for conserved.

### Neutral Model of Amino Acid Substitutions

We calculated the expected frequency of all possible amino acid substitutions generated by a single-nucleotide mutation on the basis of the codon frequencies in the sequences of interest (*D. melanogaster* group N1 or conserved) and the nucleotide transition matrix in the species. The latter was estimated from intronic SNPs in the genetic reference panel (Mackay et al. 2012). For example to calculate the frequency of lysine to glutamic acid (K→E), we considered the following changes AAA→GAA and AAG→GAG; in the first case, the expected value was the relative frequency of AAA multiplied by the relative frequency of the A→G mutation in the transition matrix and, in the second case, the relative frequency of AAG multiplied by the relative frequency of the A→G mutation in the transition matrix. To calculate the expected frequency of amino acid pairs with no information on the direction of change, we added the probabilities of the two changes; for example, for K/E, we calculated the expected frequency of K→E plus the expected frequency of E→K. The expected values were then normalized so that the total number of changes was equal to the total number of observed changes. For the comparison, we did not consider amino acid substitutions that could not be explained by a single-nucleotide mutation or amino acid substitutions that could be explained by a single-nucleotide mutation but which were not observed in proteins from the N1 class.

## Supplementary Material

msad098_Supplementary_Data

## Data Availability

Supplementary material S1, Supplementary Material online contains supplementary Tables and Figures. Supplementary material S2, Supplementary Material online is an Excel file that contains detailed information of the *S. cerevisiae* and *D. melanogaster* proteins used in the study, including their possible origin by gene duplication or de novo formation, expression levels, protein sequence properties, and SNPs, as well as GO classes. The file also contains information on the data from fig. 6, including observed and expected amino acid pairs in the alignments of proteins from two species, as well as in the alignments of proteins from three species. The program GeneBPhylo that processes OrthoFinder output is available at https://github.com/m-huertasp/GeneBPhylo. The C code for calculating the expected PN/PS given a nucleotide mutation matrix and codon frequencies table, as well as python scripts to calculate the observed and expected number of amino acid changes, are available at https://github.com/JC-therea/Montanes_J_Carlos.
