# Supplementary material for: Evolutionary Trajectories of New Duplicated and Putative De Novo Genes
Source: Mol Biol Evol. 2023 May 4;40(5):msad098. doi: 10.1093/molbev/msad098 (PMC10182736; doi:10.1093/molbev/msad098)
Supplement: msad098_Supplementary_Data [file msad098_supplementary_data.zip › Montanes_Supfile1.pdf]

Montañés et al. Evolutionary trajectories of new duplicated and putative *de novo* genes.
Supplementary Tables and Figures.

Outgroup species
Escherichia coli
Bacillus subtilus
Nostoc punctiforme
Actinobacteria bacterium
Betaproteobacteria bacterium
Entamoeba invadens
Plasmodium falciparum
Leishmania major
Arabidopsis thaliana
Zea mays
Oryza sativa
Caenorhabditis elegans
Nematostella vectensis
Daphnia pulex
Homo sapiens
Gallus gallus
Sacharomyces cerevisiae
Schizosaccharomyces pombe
Drosophila melanogaster
Tribolium castaneum

Table S1. Species used for distant homology detection. Proteins initially classified as having a putative *de novo* origin in branches N0-N5 of the yeast tree, or N0-N6 of the fly tree, and which had putative homologues in at least two of these species were discarded. Homology was assessed using BLASTP with an E-value cut-off of E-value < 0.001. *S. cerevisiae* and *S. pombe* were used when searching for distant homologues of *D. melanogaster* proteins, and *D. melanogaster* and *T. castaneum* when searching for distant homologues of *S. cerevisiae* proteins.

Species	Class	Branch Length	*de novo* events	*de novo* proteins (expression 0.1/0.5 TPM cut-off)	duplication events	duplicated proteins (expression 0.1/0.5 TPM cut-off)	*de novo* subs. duplicated
S. cerevisiae	N0	0.043	**175**	192 (175/138)	**132**	180 (171/155)	26
S. paradoxus	N1	0.025	**8**	9 (9/7)	**9**	8 (8/8)	0
S. mikatae	N2	0.026	**4**	4 (4/4)	**15**	16 (16)	0
S. kudriavzevii	N3	0.025	**14**	20 (19/18)	**27**	19 (19/18)	11
S. bayanus	N4	0.346	**72**	75 (73/70)	**170**	99 (99/97)	6
S. castellii	N5	0.168	**31**	63 (63/62)	**164**	44 (44/44)	35

Table S2. Number of events and proteins from different classes in yeast. Estimated gene birth events in different branches of the yeast tree and the corresponding *S. cerevisiae* proteins. Branch length refer to number of amino acids substitutions per site, it was calculated by Orthofinder with the IQ-tree method. 'expression' refers to the number of proteins encoded by genes with expression evidence (TPM > 0.1 or TPM > 0.5) using RNA-Seq data of *S. cerevisiae* grown in a rich medium (Blevins et al., 2021). '*de novo* sub. duplicated' indicates the number of putative *de novo* proteins originated in a given branch which underwent further duplications (in the same or in another branch).

CLADE	CLASS	GROUP	N	MIN	MEDIAN	MAX	SD
YEAST	N0	putative de novo	166	16	66	387	54.9
YEAST	N1 - N3	putative de novo	22	28	130.5	1140	247.7
YEAST	N4 - N5	putative de novo	97	37	223	1188	187.1
YEAST	Conserved	-	3054	51	435	4910	389.9
YEAST	N0	Duplication	123	36	437	1859	486.3
YEAST	N1 - N3	Duplication	35	150	468	1579	269.8
YEAST	N4 - N5	Duplication	130	72	529	2123	352.4
YEAST	Conserved	-	3054	51	435	4910	389.9
FLIES	N0	putative de novo	122	11	83.5	795	110.3
FLIES	N1 - N2	putative de novo	428	22	124	16223	803.7
FLIES	N3 - N4	putative de novo	226	28	158.5	1893	250.7
FLIES	N5 - N6	putative de novo	1431	32	223	3441	306.3
FLIES	Conserved	-	7100	40	451	18468	633.7
FLIES	N0	Duplication	223	63	256	1525	253.6
FLIES	N1 - N2	Duplication	453	61	394	2189	314.0
FLIES	N3 - N4	Duplication	168	64	370.5	1833	270.2
FLIES	N5 - N6	Duplication	1184	61	412	8255	413.8
FLIES	Conserved	-	7100	40	451	18468	633.7

Table S3. Estimated number of proteins, and their protein length, for different groups. The numbers of proteins in each class are indicated in column "N". For each class, minimum (MIN), median (MEDIAN), maximum value (MAX) and standard deviation (SD) of protein length are indicated. Conserved was a control set of proteins not involved in *de novo* gene birth events or duplications in any of the branches considered here.

Species	Class	Branch length	***de novo* events**	***de novo* proteins**	**duplication events**	**duplicated proteins**	***de novo* subs. duplicated**
S. cerevisiae	N0	0.043	**394**	410	**136**	186	**25**
S. paradoxus	N1	0.025	**17**	19	**8**	6	**0**
S. mikatae	N2	0.026	**13**	27	**21**	16	**14**
S. kudriavzevii	N3	0.025	**23**	29	**36**	21	**12**
S. bayanus	N4	0.347	**78**	83	**172**	100	**9**
S. castellii	N5	0.169	**30**	62	**162**	42	**35**

Table S4. Estimated number of events and proteins from different classes using annotations as well as RNAseq and Riboseq data in yeast. Branch length refer to number of amino acids substitutions per site, it was calculated by Orthofinder with the IQ-tree method. '*de novo* subs. duplicated' indicates the number of putative *de novo* proteins that originated in a given branch which underwent further duplications (in the same or subsequent branch).

Species	Class	Branch length	*de novo* events	***de novo*** **proteins (expression 0.1/0.5 TPM cut-off)**	duplication events	duplicated proteins (expression)	de novo subs. duplicated
D. melanogaster	N0	0.011	127	132 (89/77)	205	316(247/219)	10
D. simulans and D. sechellia	N1	0.014	92	115(90/84)	54	94(80/78)	28
D. erecta and D. yakuba	N2	0.104	351	363(320/291)	353	573(481/420)	22
D. pseudoobscura and D. persimilis	N3	0.044	144	154(121/107)	70	99(77/66)	17
D. willistoni	N4	0.035	99	111(88/75)	85	124(103/94)	22
D. grimshawi, D. mojavensis and D. virilis	N5	0.236	820	920(751/764)	500	783(720/678)	150
B. dorsalis and C. capitata	N6	0.440	731	885(699/565)	355	512(469/434)	224

Table S5. Estimated number of events and proteins from different classes in flies. Species is used to indicate the species considered to define the branches. Branch length refer to number of amino acids substitutions per site, it was calculated by Orthofinder with the IQ-tree method. '*de novo* subs. duplicated' indicates the number of putative *de novo* proteins originated in a given branch which underwent further duplications (in the same or subsequent branch). 'expression' refers to the number of genes for which we could validate expression in 3-10 days flies using RNA-Seq data from Zhang et al. (2018).

Species	Class	Branch length	***de novo* events**	***de novo* proteins**	**duplication events**	**duplicated proteins**	***de novo* subs. duplicated**
D. melanogaster	N0	0.011	162	185	208	325	32
D. simulans and *D. sechellia*	N1	0.014	80	87	52	92	10
D. erecta and *D. yakuba*	N2	0.104	383	399	352	577	30
D. pseudoobscura and *D. persimilis*	N3	0.044	126	132	73	103	13
D. willistoni	N4	0.035	129	147	94	141	33
D. grimshawi, D. mojavensis and *D. virilis*	N5	0.236	827	909	492	768	129
B. dorsalis and *C. capitata*	N6	0.440	730	904	357	522	248

Table S6. Estimated number of events and proteins from different classes using annotations as well as RNAseq and Riboseq data in flies. Estimated gene birth events in different branches of the yeast tree and the corresponding *D. melanogaster*. Branch length refer to number of amino acids substitutions per site, it was calculated by Orthofinder with the IQ-tree method. '*de novo* subs. duplicated' indicates the number of putative *de novo* proteins that originated in a given branch which underwent further duplications (in the same or subsequent branch).

Clade	Class	Group	n	PN_o	PS_o	PN_e	PS_e	PN_o/ PS_O	PN_e/ PS_E	PN/PS	pvalue	PN/PS mean samp.	PN/PS SD samp.
yeast	Conserv.	-	3220	10750	28361	27951.4	11159.6	0.38	2.5	0.15	0	0.151	0.004
yeast	N0	putative de novo	150	292	145	314.21	122.79	2.01	2.56	0.78	0.1196	0.806	0.134
yeast	N1 - N4	putative de novo	90	431	395	592.38	233.62	1.09	2.54	0.43	4.38E-16	0.429	0.061
yeast	N5	putative de novo	28	120	116	169.56	66.44	1.03	2.55	0.409	4.44E-06	0.407	0.087
yeast	N0	Duplication	114	612	947	1112.52	446.48	0.65	2.49	0.26	2.20E-72	0.262	0.047
yeast	N1 - N4	Duplication	118	638	1349	1417.51	569.49	0.47	2.49	0.19	7.61E-135	0.191	0.025
yeast	N5	Duplication	44	213	438	465.44	185.56	0.49	2.51	0.20	3.18E-44	0.198	0.035
flies	Conserv.	-	8800	35305	129721	122113	42912.6	0.27	2.85	0.092	0	0.096	0.002
flies	N0	putative de novo	122	272	253	381.91	143.09	1.08	2.67	0.40	4.08E-12	0.413	0.078
flies	N1 - N2	putative de novo	428	1784	1545	2481.03	847.97	1.15	2.93	0.39	1.06E-70	0.396	0.031
flies	N3 - N4	putative de novo	226	1229	1448	1989.06	687.94	0.85	2.89	0.29	1.32E-99	0.293	0.031
flies	N5	putative de novo	769	3577	5476	6711.96	2341.04	0.65	2.87	0.23	0	0.228	0.013
flies	N6	putative de novo	661	2862	6064	6603.08	2322.92	0.47	2.84	0.17	0	0.167	0.012
flies	N0	Duplication	220	251	381	469.33	162.67	0.66	2.89	0.23	5.06E-35	0.238	0.055
flies	N1 - N2	Duplication	452	2792	5485	6164.73	2112.27	0.51	2.92	0.17	0	0.175	0.012
flies	N3 - N4	Duplication	168	914	2148	2272.89	789.11	0.43	2.88	0.15	2.53E-264	0.148	0.019
flies	N5	Duplication	697	2829	9821	9378.5	3271.5	0.29	2.87	0.10	0	0.1	0.006
flies	N6	Duplication	483	1510	6611	6003.05	2117.95	0.23	2.83	0.081	0	0.081	0.006

Table S7. Synonymous and non-synonymous substitutions in yeast and flies. Number of observed synonymous (PS_O) and non-synonymous (PN_O) single nucleotide polymorphisms (SNPs) and expected values of synonymous (PS_E) and non-synonymous (PN_E) SNPs under neutrality, for different groups of coding sequences. N indicates the number of proteins analyzed for each class. Conserv. refers to proteins with homologues outside the clade and which have not undergone duplications in the clade. The column PVALUE indicates the p-value of the χ^2 test between observed and expected values where the null hypothesis is no difference between observed values and expected values. PN/PS is the result of dividing PN_O/PS_O by PN_E/PS_E; it is expected to be 1 in the absence of selection and the closer this value is to 0 the stronger the purifying selection. SN mean samp. SN SD samp indicate the mean ratio and standard deviation between SNo and SNe (SNo/SNe) over 1000 iterations using 1/3 of all the proteins at any given class.

Change	*D. sech* N1	*D. sech* conserv.	*D. sim* N1	*D. sim* conserv.	FDR *D. sech*	FDR *D. sim*
DK	11	169	1	193	6.97E-05	1
DF	6	49	1	36	0.00031405	0.87764386
DE	30	5412	28	6326	0.00096449	5.66E-07
DS	12	342	2	266	0.00208301	1
EK	35	1853	29	2080	0.00238529	0.66997576
AE	2	994	10	1153	0.0290362	1
EQ	9	1785	13	2056	0.09009851	0.66997576
DI	4	98	1	65	0.09009851	0.97849118
EM	3	57	1	56	0.09009851	0.97849118
EY	3	59	0	52	0.09009851	1
DT	5	194	1	169	0.16263483	1
EH	2	108	2	105	0.9170999	0.87764386
DN	17	2036	18	2218	0.92956662	0.97849118
EG	15	1236	19	1295	0.92956662	0.66997576
AD	5	769	6	808	0.92956662	1
DR	2	133	0	64	0.92956662	1
DL	1	69	1	69	0.92956662	0.97849118
EI	1	63	0	85	0.92956662	1
EP	0	144	0	140	0.92956662	1
DQ	0	160	0	162	0.92956662	0.97849118
DG	11	1273	7	1260	1	0.66997576
EV	4	585	2	738	1	0.66997576
ER	4	325	4	200	1	0.66997576
DV	4	414	6	446	1	0.97849118
DY	3	252	4	216	1	0.71349501
DH	3	338	4	339	1	1
ES	3	323	5	281	1	0.71349501
EN	2	299	2	292	1	1
ET	2	165	2	213	1	1
DP	1	113	0	112	1	1
EL	1	111	2	148	1	1
DM	0	45	0	15	1	1
CE	0	25	0	12	1	1
DW	0	6	0	5	1	1
CD	0	38	1	22	1	0.71349501
EF	0	37	0	27	1	1
EW	0	10	1	14	1	0.66997576

Table S8. Amino acid changes involving acidic residues in pairwise alignments of proteins from groups N1 and conserved. The alignments are *D. melanogaster* and *D. sechellia* (indicated as *D. sech*) or *D. melanogaster* and *D. simulans* (indicated as *D. sim).* The column change indicates the amino acids involved (one letter symbol). FDR columns indicate the adjusted p-value (by false discovery rate method) of Fisher's test comparing the frequency of each specific change between N1 and conserved proteins.

Organism	Genome link	Annotation link	SNPs	Proteins	Filtered	No WGD
Saccharomyces cerevisiae	https://downloads.FUNGIgenome.org/sequence/S288C_reference/genome_releases/S288C_reference_genome_R64-2-1_20150113.tgz	https://doi.org/10.6084/m9.figshare.7851521.v2	http://1002genomes.u-strasbg.fr/files/	5802	5785	4770
Saccharomyces paradoxus	http://www.saccharomycessensustricto.org/current/Spar/Spar.ultrascaf%22%20%5Ct%20%22_blank	https://doi.org/10.6084/m9.figshare.7851521.v2	-	5087	5078	
Saccharomyces mikatae	http://www.saccharomycessensustricto.org/current/Smik/Smik.ultrascaf%22%20%5Ct%20%22_blank	https://doi.org/10.6084/m9.figshare.7851521.v2	-	5119	5113	
Saccharomyces kudriavzevii	http://www.saccharomycessensustricto.org/current/Skud/Skud.ultrascaf%22%20%5Ct%20%22_blank	https://doi.org/10.6084/m9.figshare.7851521.v2	-	5074	5068	
Saccharomyces bayanus	http://www.saccharomycessensustricto.org/current/Sbay/Sbay.ultrascaf%22%20%5Ct%20%22_blank	https://doi.org/10.6084/m9.figshare.7851521.v2	-	5065	5049	
Naumovozyma castellii	https://www.ncbi.nlm.nih.gov/genome/68?genome_assembly_id=22766	https://doi.org/10.6084/m9.figshare.7851521.v2	-	5587	5587	
Lachancea kluyveri	http://www.genolevures.org/download/sequence/chromosome/Sakl0A_contig.fasta%22%20%5Ct%20%22_blank	https://doi.org/10.6084/m9.figshare.7851521.v2	-	5013	4907	
Lachancea thermotolerans	https://www.ncbi.nlm.nih.gov/genome/208?genome_assembly_id=28553	https://doi.org/10.6084/m9.figshare.7851521.v2	-	5091	5091	
Lachancea waltii	http://ygob.ucd.ie/ygob/data/v7-Aug2012/Lwaltii_sequence.fsa%22%20%5Ct%20%22_blank	https://doi.org/10.6084/m9.figshare.7851521.v2	-	5051	5038	
Kluyveromyces lactis	https://www.ncbi.nlm.nih.gov/genome/?term=txid28985[orgn]	https://doi.org/10.6084/m9.figshare.7851521.v2	-	5078	5075	
Schizosaccharomyces pombe	https://www.ncbi.nlm.nih.gov/genome/14?genome_assembly_id=22534	https://doi.org/10.6084/m9.figshare.7851521.v2	-	5119	5114	

Table S9. Proteomes and associated data for yeast. The source of the reference genome and annotations is indicated. SNPs refers to the source of single nucleotide polymorphisms in the reference species. Proteins refers to the initial number of protein sequences recovered from the databases. Filtered refers to the selection of proteins after eliminating genes with at least a 10% overlap with another gene and taking the longest isoform for each gene. No WGD is when proteins originated from the whole genome duplication before the Saccharomyces diversification were discarded.

Organism	Genome link	Annotation link	SNPs	Proteins	Filtered
Drosophila melanogaster	ftp://ftp.flybase.net/genomes/Drosophila_melanogaster/dmel_r6.01_FB2014_04/fasta/dmel-all-chromosome-r6.01.fasta.gz	ftp://ftp.flybase.net/genomes/Drosophila_melanogaster/dmel_r6.01_FB2014_04/gff/dmel-all-r6.01.gff.gz	ftp://ftp.hgsc.bcm.edu/DGRP/freeze2_Feb_2013/liftover_data_for_D.mel6.0_from_William_Gilks_Oct_2015/DGRP_liftover_Oct2015/dgrp2_dm6.vcf.gz	29706	13533
Drosophila yakuba	ftp://ftp.flybase.net/genomes/Drosophila_yakuba/dyak_r1.05_FB2016_05/fasta/dyak-all-chromosome-r1.05.fasta.gz	ftp://ftp.flybase.net/genomes/Drosophila_yakuba/dyak_r1.05_FB2016_05/gff/dyak-all-filtered-r1.05.gff.gz	-	22321	13839
Drosophila pseudoobscura	ftp://ftp.flybase.net/genomes/Drosophila_pseudoobscura/dpse_r3.04_FB2018_05/fasta/dpse-all-chromosome-r3.04.fasta.gz	ftp://ftp.flybase.net/genomes/Drosophila_pseudoobscura/dpse_r3.04_FB2018_05/gff/dpse-all-r3.04.gff.gz	-	22786	13901
Drosophila virilis	ftp://ftp.flybase.net/genomes/Drosophila_virilis/dvir_r1.07_FB2018_05/fasta/dvir-all-chromosome-r1.07.fasta.gz	ftp://ftp.flybase.net/genomes/Drosophila_virilis/dvir_r1.07_FB2018_05/gff/dvir-all-r1.07.gff.gz	-	19865	12937
Drosophila grimshawi	ftp://ftp.flybase.net/genomes/Drosophila_grimshawi/dgri_r1.3_FB2016_02/fasta/dgri-all-chromosome-r1.3.fasta.gz	ftp://ftp.flybase.net/genomes/Drosophila_grimshawi/dgri_r1.3_FB2016_02/gff/dgri-all-r1.3.gff.gz	-	14676	14672
Drosophila simulans	ftp://ftp.flybase.net/genomes/Drosophila_simulans/dsim_r2.02_FB2017_04/fasta/dsim-all-chromosome-r2.02.fasta.gz	ftp://ftp.flybase.net/genomes/Drosophila_simulans/dsim_r2.02_FB2017_04/gff/dsim-all-r2.02.gff.gz	-	23343	13443
Drosophila sechellia	ftp://ftp.flybase.net/genomes/Drosophila_sechellia/dsec_r1.3_FB2016_05/fasta/dsec-all-chromosome-r1.3.fasta.gz	ftp://ftp.flybase.net/genomes/Drosophila_sechellia/dsec_r1.3_FB2016_05/gff/dsec-all-r1.3.gff.gz	-	16126	16120
Drosophila erecta	ftp://ftp.flybase.net/genomes/Drosophila_erecta/dere_r1.3_FB2014_03/fasta/dere-all-chromosome-r1.3.fasta.gz	ftp://ftp.flybase.net/genomes/Drosophila_erecta/dere_r1.3_FB2014_03/gff/dere-all-r1.3.gff.gz	-	14917	14913
Drosophila persimilis	ftp://ftp.flybase.net/genomes/Drosophila_persimilis/dper_r1.3_FB2016_05/fasta/dper-all-chromosome-r1.3.fasta.gz	ftp://ftp.flybase.net/genomes/Drosophila_persimilis/dper_r1.3_FB2016_05/gff/dper-all-r1.3.gff.gz	-	16495	16491
Drosophila willistoni	ftp://ftp.flybase.net/genomes/Drosophila_willistoni/dwil_r1.3_FB2015_01/fasta/dwil-all-chromosome-r1.3.fasta.gz	ftp://ftp.flybase.net/genomes/Drosophila_willistoni/dwil_r1.3_FB2015_01/gff/dwil-all-r1.3.gff.gz	-	15197	15196
Drosophila mojavensis	ftp://ftp.flybase.net/genomes/Drosophila_mojavensis/dmoj_r1.3_FB2015_01/fasta/dmoj-all-chromosome-r1.3.fasta.gz	ftp://ftp.flybase.net/genomes/Drosophila_mojavensis/dmoj_r1.3_FB2015_01/gff/dmoj-all-r1.3.gff.gz	-	14408	14407
Bactrocera dorsalis	http://www.insect-genome.com/data/genome_download/Bactrocera_dorsalis/Bactrocera_dorsalis_genomic.fasta.gz	http://www.insect-genome.com/data/genome_download/Bactrocera_dorsalis/Bactrocera_dorsalis_genomic.gff3.gz	-	15880	10333
Ceratitis capitata	http://www.insect-genome.com/data/genome_download/Ceratitis_capitata/Ceratitis_capitata_genomic.fasta.gz	http://www.insect-genome.com/data/genome_download/Ceratitis_capitata/Ceratitis_capitata_genomic.gff3.gz	-	22730	13812
Anopheles gambiae	http://www.insect-genome.com/data/genome_download/Anopheles_gambiae/Anopheles_gambiae_genomic.fasta.gz	http://www.insect-genome.com/data/genome_download/Anopheles_gambiae/Anopheles_gambiae_genomic.gff3.gz	-	11871	10202
Aedes aegypti	http://www.insect-genome.com/data/genome_download/Aedes_aegypti/Aedes_aegypti_genomic.fasta.gz	http://www.insect-genome.com/data/genome_download/Aedes_aegypti/Aedes_aegypti_genomic.gff3.gz	-	15186	13922
Tribolium castaneum	http://www.insect-genome.com/data/genome_download/Tribolium_castaneum/Tribolium_castaneum_genomic.fasta.gz	http://www.insect-genome.com/data/genome_download/Tribolium_castaneum/Tribolium_castaneum_genomic.gff3.gz	-	16397	16395

Table S10. Proteomes and associated data for the flies species. The source of the reference genome and annotations is indicated. SNPs refers to the source of single nucleotide polymorphisms in the reference species. Proteins refers to the initial number of protein sequences recovered from the databases. Filtered refers to the selection of proteins after eliminating genes with at least a 10% overlap with another gene and taking the longest isoform for each gene.

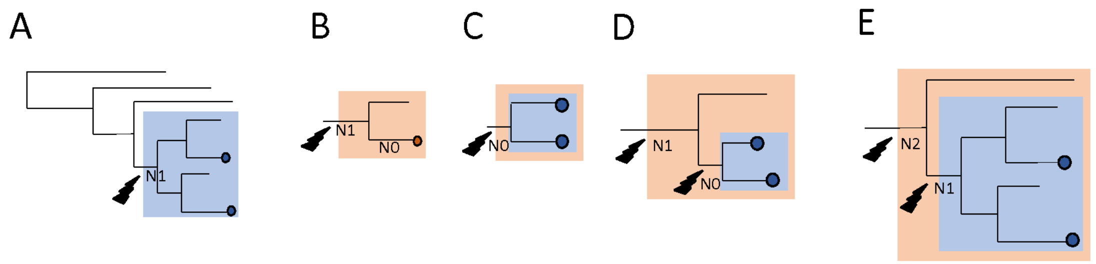

Figure S1. Examples of different scenarios of new gene birth. A. A gene duplication event at branch N1. **B**. A putative *de novo* gene originated at branch N1. **C.** A putative species-specific *de novo* gene (N0) is subsequently duplicated. **D.** A putative *de novo* gene originated at branch N1 is duplicated at branch N0. **E.** A putative *de novo* gene originated at branch N2 is subsequently duplicated at branch N1. Filled circles indicate gene copies in the reference species.

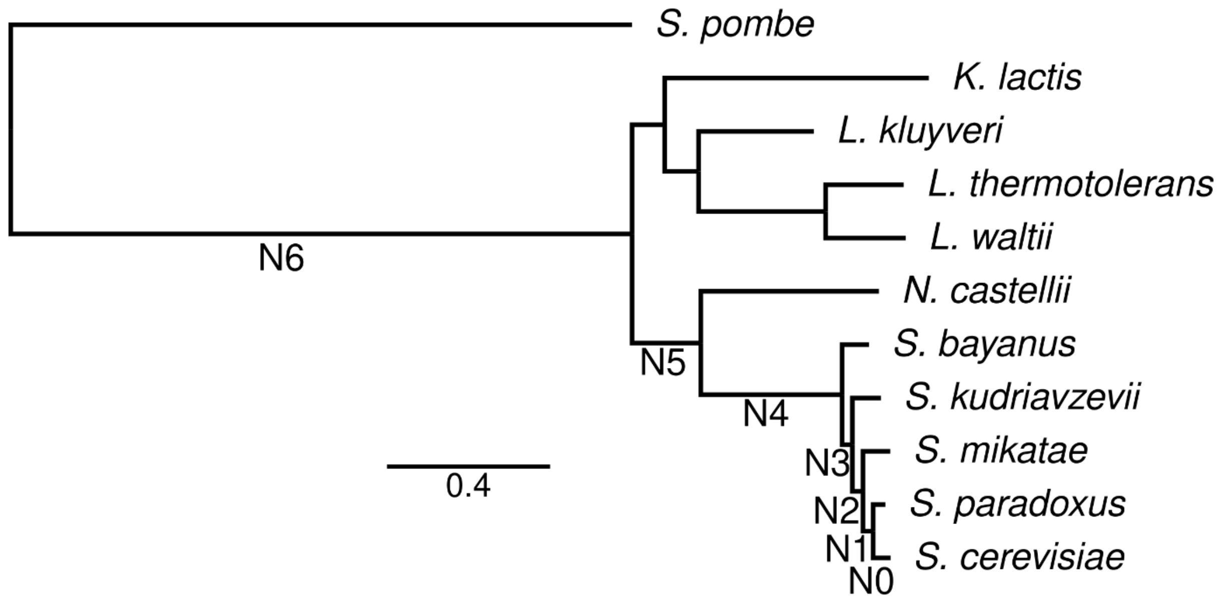

Figure S2. Species tree for yeast. Branch length is in amino acid substitutions per site. Branches were labelled starting with the reference species, *S. cerevisiae*. Gene birth events, and the resulting proteins, were studied for branches N0 to N5.

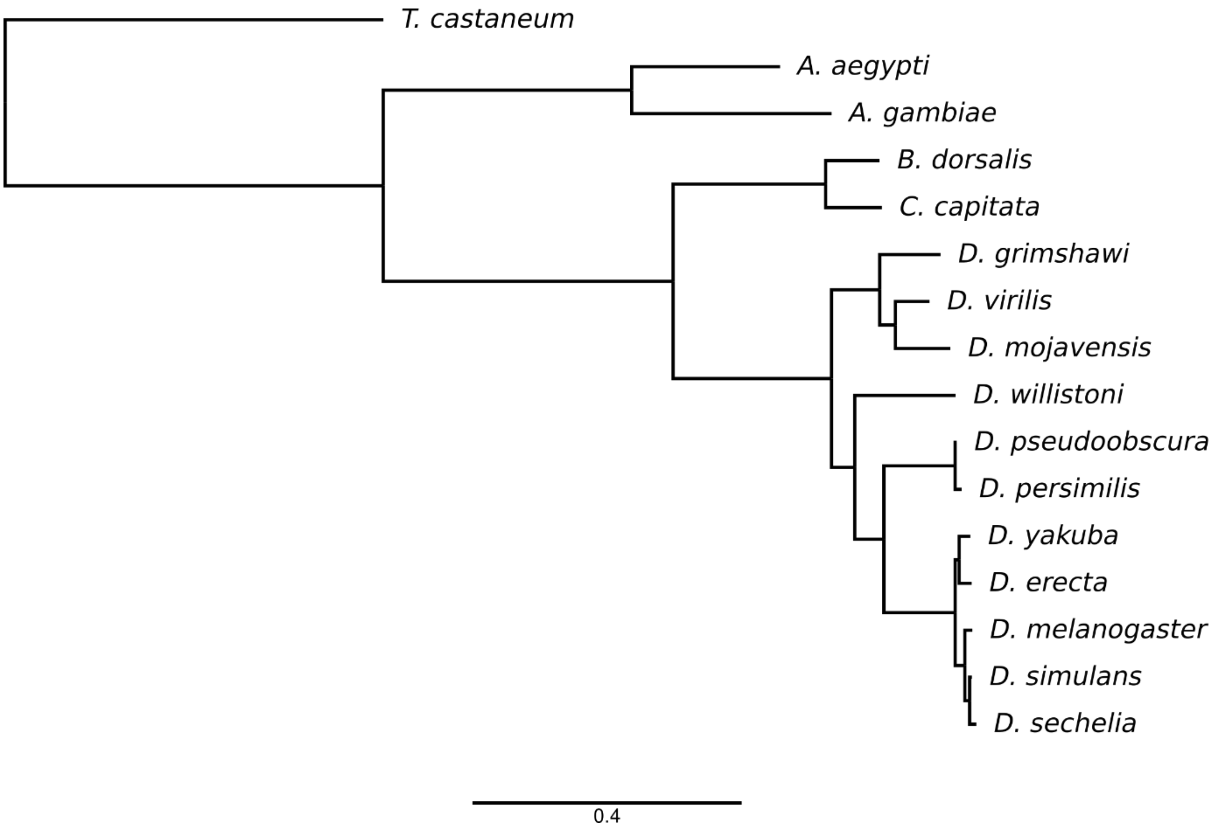

Figure S3. Species tree for flies. Branch length is in amino acid substitutions per site. Branches were labelled starting with the reference species, *D. melanogaster*. Gene birth events, and the resulting proteins, were studied for branches N0 to N6.

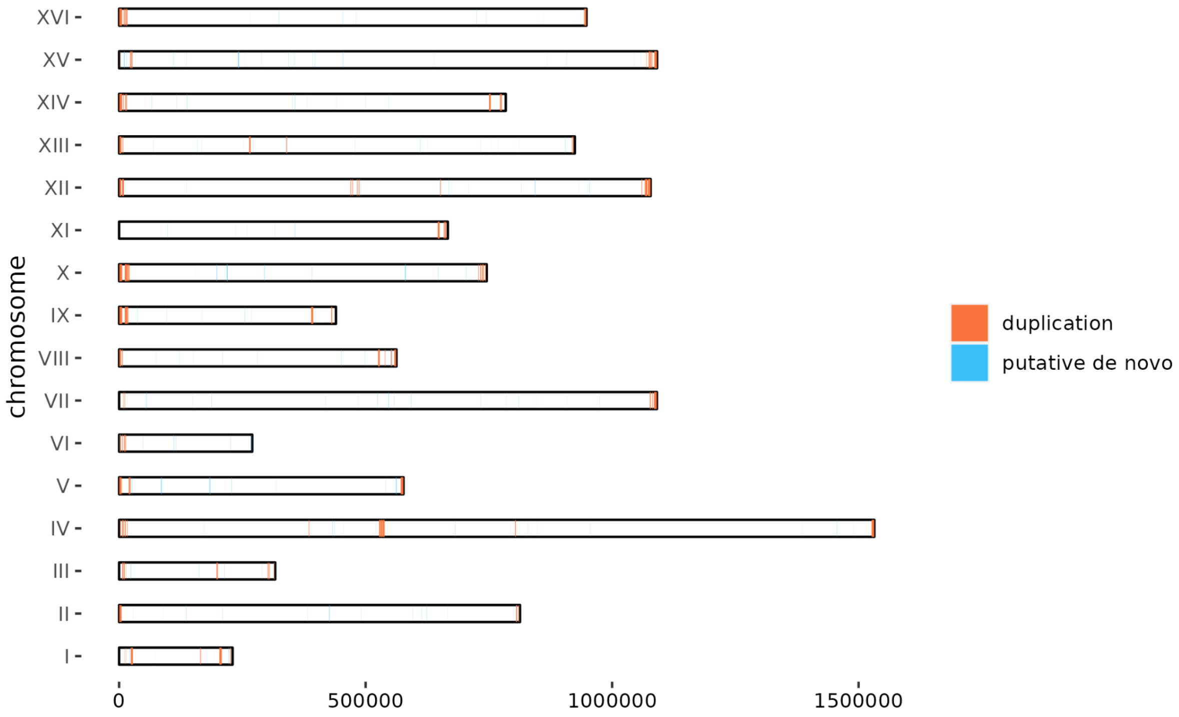

Figure S4. Location of N0 genes in the *S. cerevisiae* genome. Y-axis indicates each *S. cerevisiae* chromosome. X-axis indicates the length of each chromosome in nucleotides. Duplicated genes tend to be located in sub-telomeric regions whereas no bias is observed for putative *de novo* genes.

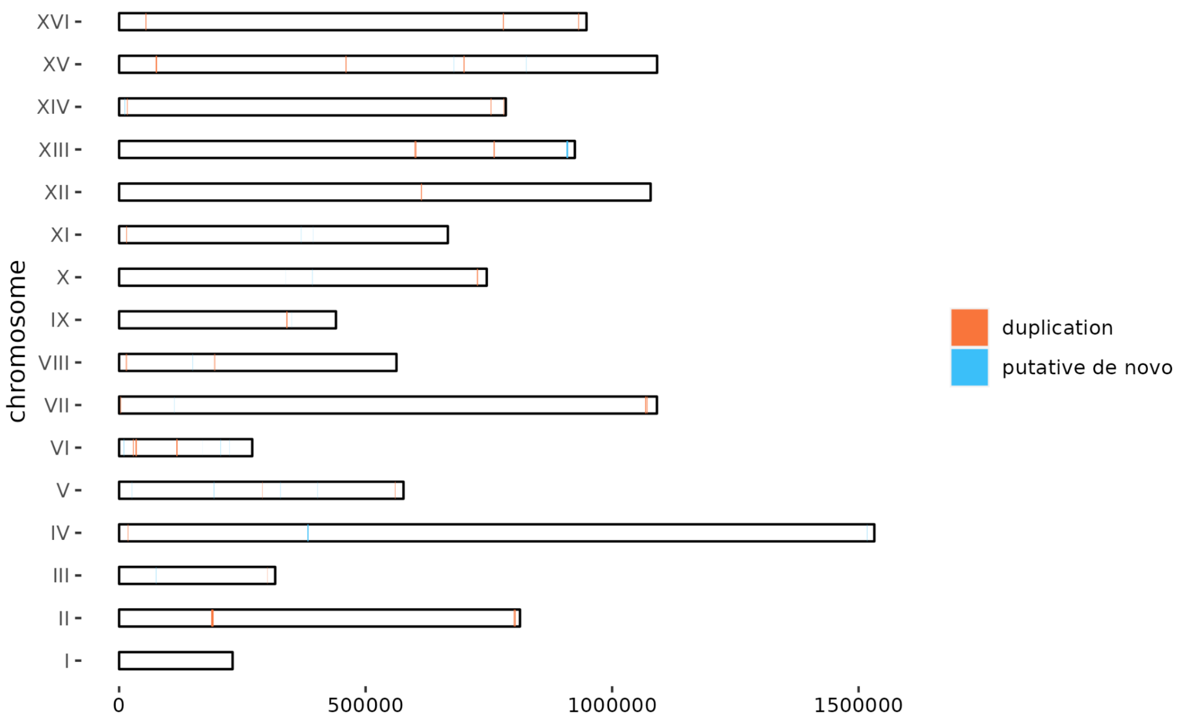

Figure S5. Location of N1-N3 genes in the *S. cerevisiae* genome. Y-axis indicates each *S. cerevisiae* chromosome. X-axis indicates the length of each chromosome in nucleotides. No bias is observed for duplicated or putative *de novo* genes.

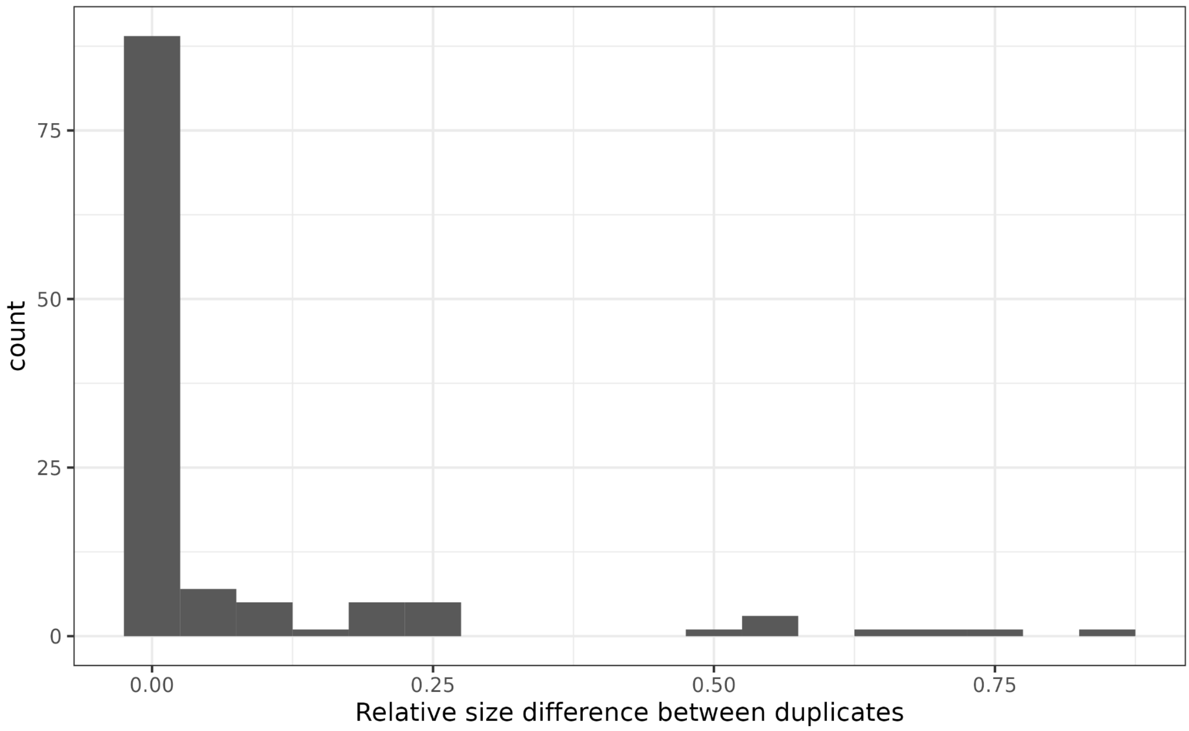

Figure S6. Comparison of the size of *D. melanogaster* duplicated proteins from N0. Shown is the relative size difference between pairs of protein duplicates originating from a given gene duplication event ((largest – shortest)/largest). The percentage of cases in which the smallest copies is less than 0.9 times the size of the largest copy is 17.5%, less than 0.8 15.2% and less than 0.75 9.2%.

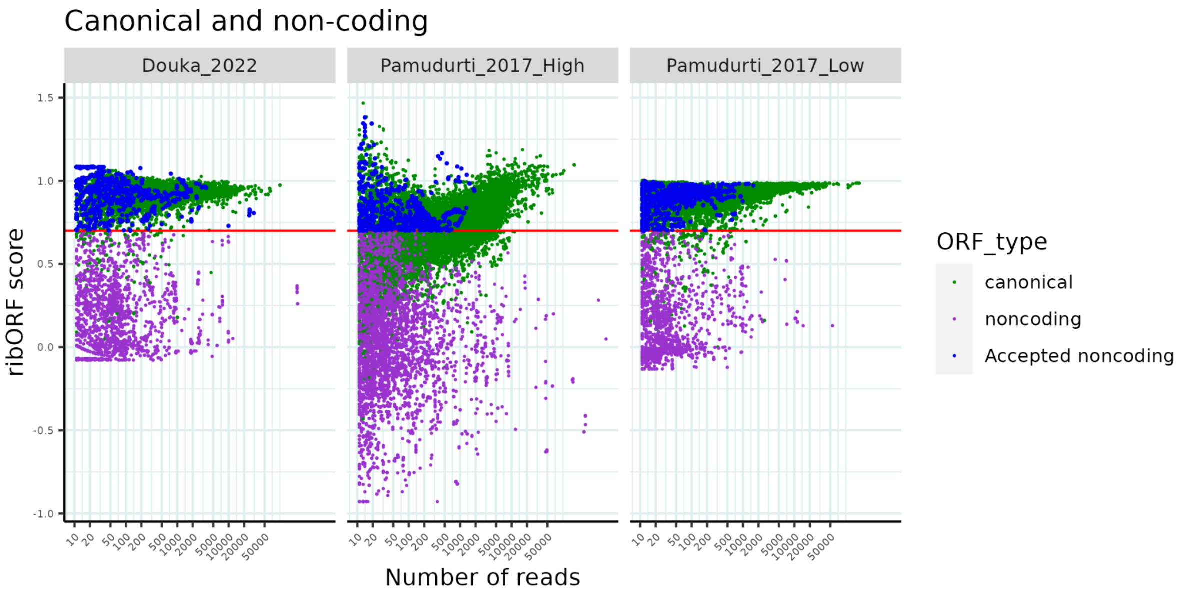

Figure S7. Prediction of novel translated ORFs in *D. melanogaster* using three different Ribo-Seq datasets. RibORF score (Y axis) and number of mapped reads (X axis) were obtained with the RibORF program (Ji et al., 2015). Douka_2022 refers to Ribo-Seq data from S2 cells, Pamudurti_2017 to data from adult fly heads (high and low detergent conditions were employed). The 'Accepted noncoding' represents putative novel translated ORFs. The cut-off to select *bona fide* translated ORFs was 11 mapped Ribo-Seq reads and a RibORF score of 0.7.

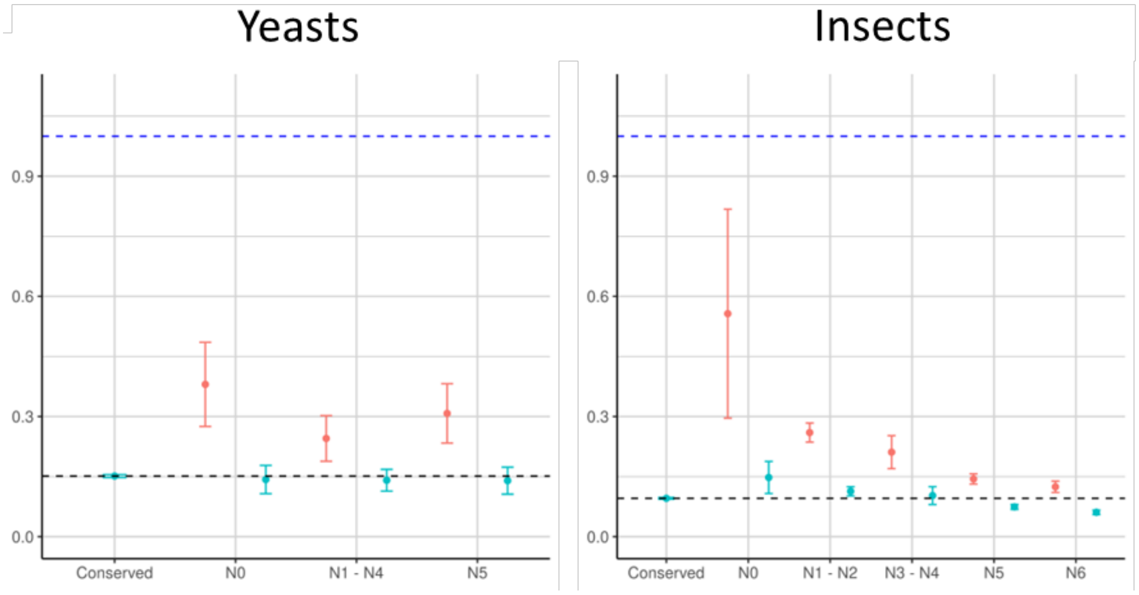

Figure S8. Purifying selection estimates for the fastest and lowest evolving gene copies. We examined the observed PN/PS of the genes originated at gene duplicated events assigned to different branches in the tree. Y axis represents the normalized ratio between non-synonymous and synonymous substitutions SNPs (PN/PS). Branches have been grouped as in Figure 4 in the manuscript. Values around 1 are expected in the absence of purifying selection. Black dashed line indicates the PN/PS (obs/exp) of using all the genes in each of the reference organisms used in this study (*S. cerevisiae*, *D. melanogaster*). Standard deviation in each point was calculated using subsampling (n = 1000) of 1/3 of the genes in each group. Red: fastest evolving gene copy on the basis of the observed PN/PS; blue: lowest evolving gene copy on the basis of the observed PN/PS. If the observed PN/PS was the same the proteins were assigned at random to classes fastest and slowest.

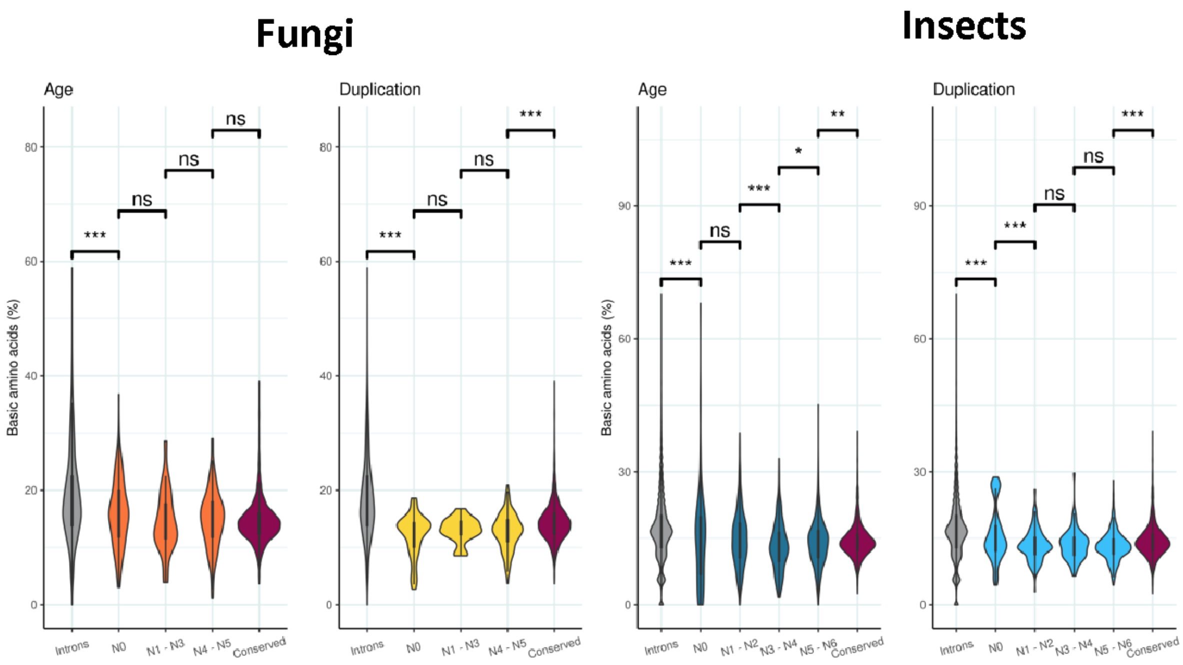

Figure S9. Frequency of positively charged residues. The relative frequency of arginine, lysine and histidine is shown for different groups of proteins and for virtually translated sequences in introns. Conserved was a control set of proteins not involved in *de novo* gene birth events or duplications in any of the branches considered here. Mann-Whitney-Wilcoxon tests were performed to compare contiguous groups in the graph; significance is denoted as * p-value < 0.05; ** p-value < 10-2; *** p-value < 10-3; ns: non-significant.

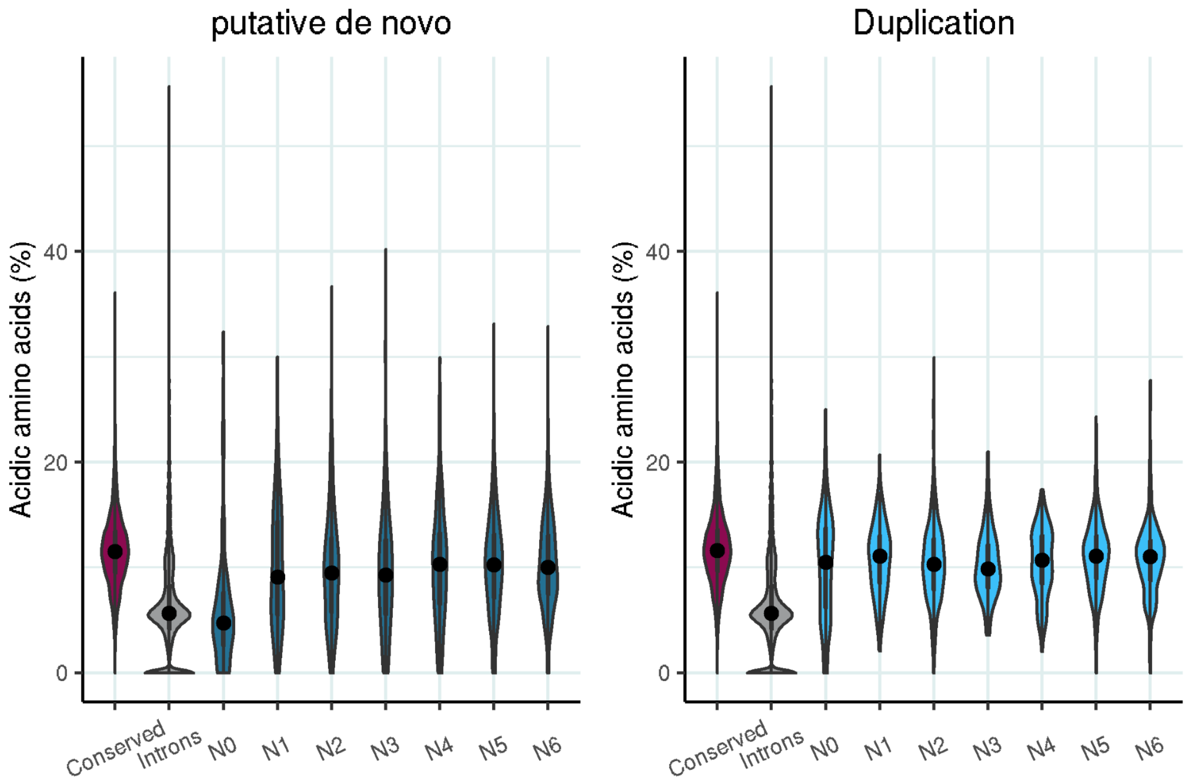

Figure S10. Proportion of negatively charged amino acids in *D. melanogaster* proteins. Data is shown for the complete set of branches, for putative *de novo* and duplicated proteins. Putative *de novo* proteins at N0 have a similar frequency of acid amino acids as virtually translated non-coding sequences (Introns). Conserved was a control set of proteins not involved in *de novo* gene birth events or duplications in any of the branches considered here.

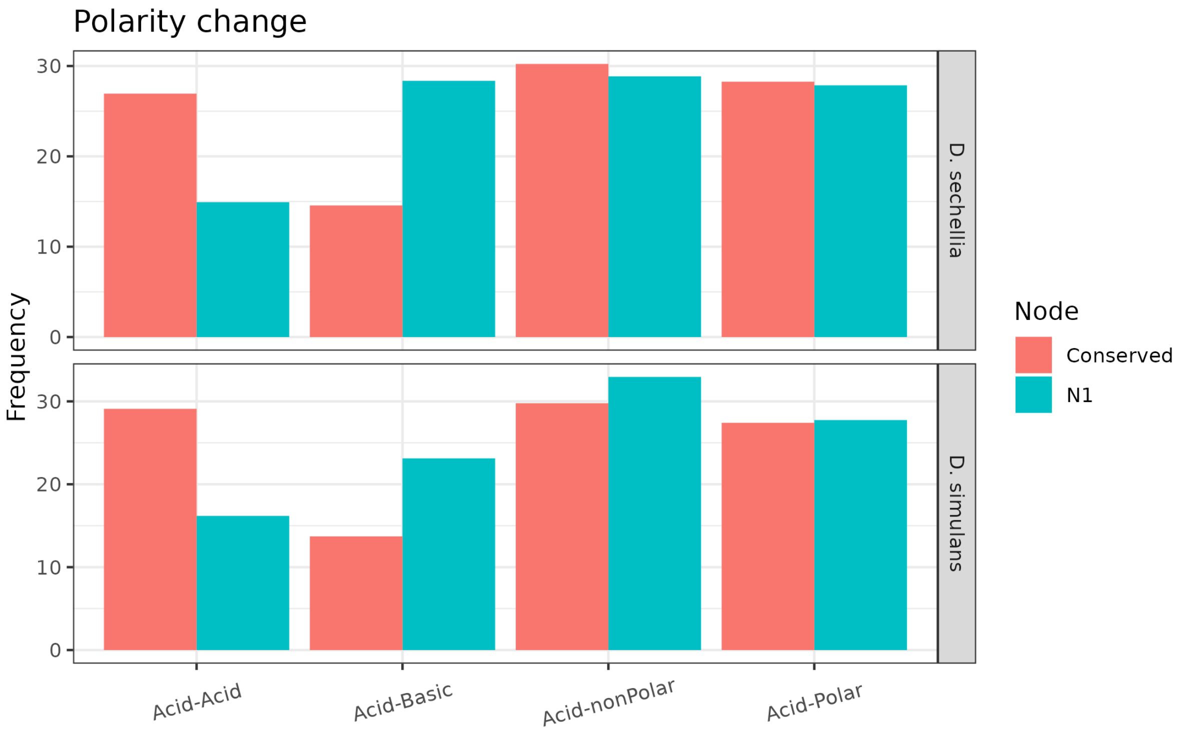

Figure S11. Comparison of different types of pairs amino acids. The changes were retrieved from pairwise sequence alignments *D. melanogaster-D. simulans* and *D. melanogaster – D. sechellia*. Acidic to acidic substitutions (D to E or *vice versa*) were significantly underrepresented in both cases (FDR < 10^{-3}). Instead, several basic to acidic substitutions were significant in comparisons between *D. melanogaster* and *D. sechellia* (E/K FRD = 0.0024, D/K FDR=0.00007).

from/to	A	C	G	T
A	0	46247	101347	74995
C	55021	0	35614	117275
G	116998	35607	0	55342
T	75316	101451	46050	0

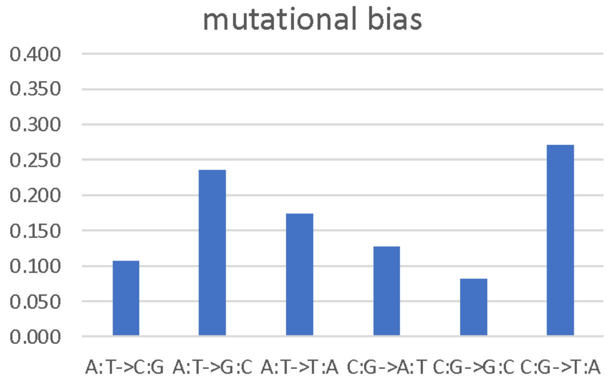

Figure S12. Mutational bias in *Drosophila melanogaster* derived from intronic SNPs. The SNPs were obtained from 192 inbred strains derived from a single outbred population of *D. melanogaster* (Mackay et al. 2012). The table on the left shows the number of SNPs in the intronic regions, for example we counted 55,021 SNPs in which C was mutated to A.
